# The Energy Landscape of Human Serine Racemase

**DOI:** 10.3389/fmolb.2018.00112

**Published:** 2019-01-09

**Authors:** Samanta Raboni, Marialaura Marchetti, Serena Faggiano, Barbara Campanini, Stefano Bruno, Francesco Marchesani, Marilena Margiotta, Andrea Mozzarelli

**Affiliations:** ^1^Department of Food and Drug, University of Parma, Parma, Italy; ^2^Department of Drug Science and Technology, University of Turin, Turin, Italy; ^3^Institute of Biophysics, National Research Council, Pisa, Italy; ^4^National Institute of Biostructures and Biosystems, Rome, Italy

**Keywords:** pyridoxal 5′-phosphate, enzyme catalysis, allosteric regulation, conformational landscape, D-serine, N-methyl-D-aspartate receptor, neuropathologies

## Abstract

Human serine racemase is a pyridoxal 5′-phosphate (PLP)-dependent dimeric enzyme that catalyzes the reversible racemization of L-serine and D-serine and their dehydration to pyruvate and ammonia. As D-serine is the co-agonist of the N-methyl-D-aspartate receptors for glutamate, the most abundant excitatory neurotransmitter in the brain, the structure, dynamics, function, regulation and cellular localization of serine racemase have been investigated in detail. Serine racemase belongs to the fold-type II of the PLP-dependent enzyme family and structural models from several orthologs are available. The comparison of structures of serine racemase co-crystallized with or without ligands indicates the presence of at least one open and one closed conformation, suggesting that conformational flexibility plays a relevant role in enzyme regulation. ATP, Mg^2+^, Ca^2+^, anions, NADH and protein interactors, as well as the post-translational modifications nitrosylation and phosphorylation, finely tune the racemase and dehydratase activities and their relative reaction rates. Further information on serine racemase structure and dynamics resulted from the search for inhibitors with potential therapeutic applications. The cumulative knowledge on human serine racemase allowed obtaining insights into its conformational landscape and into the mechanisms of cross-talk between the effector binding sites and the active site.

## Introduction

The N-methyl-D-aspartate (NMDA) receptors are ligand-gated ion channels involved in synapse formation, synaptic plasticity, learning and memory potentiation (Paoletti et al., 2013). They are the only neurotransmitter receptors whose activation requires two distinct agonists, glutamate and either glycine or D-serine, with the latter ones sharing the same binding site. Intermediates of the kynurenine pathway also bind the NMDA receptors: quinolinic acid is a strong agonist, whereas kynurenic acid acts as an antagonist (Németh et al., 2006; Lugo-Huitrón et al., 2013).

Increased levels of D-serine are associated with neuronal excitotoxicity caused by excess influx of calcium ions, as observed in several pathological conditions, including Parkinson and Alzheimer diseases, stroke and amyotrophic lateral sclerosis. On the other hand, low levels of D-serine are associated with schizophrenia. These pathological conditions prompted the search for the enzyme responsible for the production of D-serine in the brain. Serine racemase (SR) was first localized in astrocytes and later in neurons. Nowadays, the prevalent view is that the main production of D-serine takes place in neurons (Wolosker et al., 2016). Then, D-serine is exported via the ASC-1 transporter to astrocytes, where it is stored and subsequently released in the synaptic space (Wolosker et al., 2016). Other D-amino acids, such as D-aspartate and D-alanine, were detected in the brain (Hashimoto et al., 1992). The racemases involved in their production have not yet been identified (Conti et al., 2011). SR itself was proposed to be responsible for the production of D-aspartate, an agonist of the NMDA receptors (Ito et al., 2016).

SR activity depends on the coenzyme pyridoxal 5′-phosphate (PLP), in contrast with other racemases that are PLP-independent (Conti et al., 2011). A unique feature of human SR (hSR) is the modulation of its activity by several ligands, protein interactors and post-translational modifications (PTMs), suggesting significant conformational plasticity. In this review, we will present SR structure, dynamics, function and regulation, with special emphasis on the human ortholog, discussing them in the frame of the enzyme energy landscape.

## SR Structure

**Figure 1:**
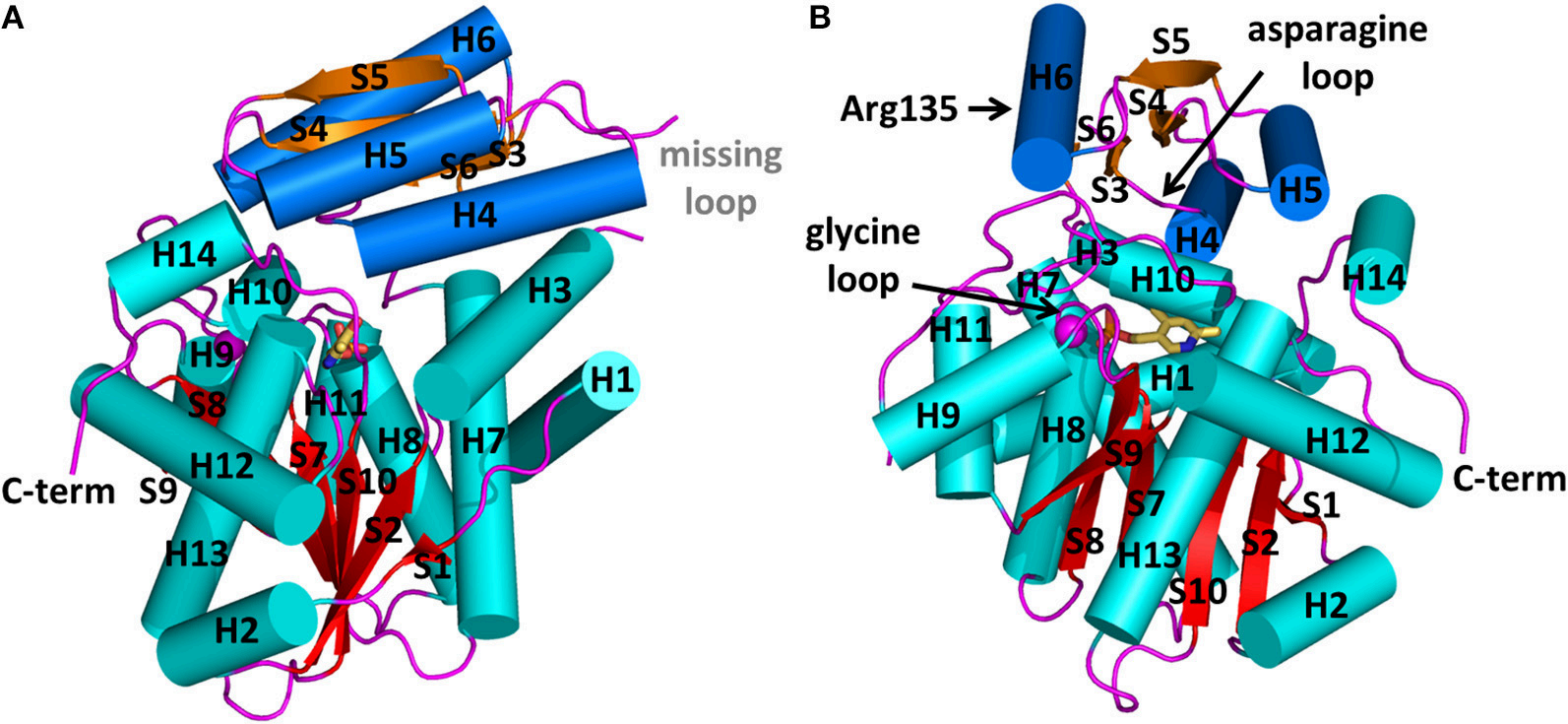
Structure of human serine racemase (PDB code 3L6B) shown in two orthogonal views **(A,B)**. α-helices and β-strands belonging to the large domain are colored in cyan and red, respectively, while those belonging to the small domain are reported in blue and orange, respectively. The divalent cation is represented as a pink sphere. All loops are colored in pink. The positions of the asparagine and glycine loops and of Arg135 are indicated by arrows.

PLP-dependent enzymes are classified in seven fold types (Grishin et al., 1995; Jansonius, 1998; Mehta and Christen, 2000; Schneider et al., 2000; Percudani and Peracchi, 2009). Traditional classification of PLP-dependent enzymes assigns fold-type I to the aspartate aminotransferase family, the largest and best characterized family (Bruno et al., 2001; Phillips et al., 2002; Kaiser et al., 2003; Storici et al., 2004; Spyrakis et al., 2014). Fold type II group includes tryptophan synthase, *O*-acetylserine sulfhydrylase, threonine deaminase and serine dehydratase (Bettati et al., 2000; Campanini et al., 2003; Raboni et al., 2003, 2005, 2007, 2009, 2010; Spyrakis et al., 2006, 2013). Bacterial alanine racemase is the archetypal protein of fold type III and the D-amino acid aminotransferase family is the most representative example of the fold type IV subgroup. Glycogen phosphorylase corresponds to fold type V. Fold types VI and VII were more recently introduced and include D-lysine 5,6-aminomutase and lysine 2,3-aminomutase, respectively. SR exhibits the type II fold, which consists of a large and a small domain with similar α/β architecture, constituted of a central β-sheet surrounded by helices. The PLP cofactor, covalently bound to a lysine of the large domain, lies in a cleft between the two domains (Figures 1A,B).

At present, 10 SR X-ray crystallographic structures have been deposited in the PDB, and, among these, three structures are of the human enzyme (Table 1). In hSR numbering, the large domain is formed by residues 1–68 and 157–340, while the small domain comprises residues between 78 and 155. The longer connecting region, residues 69–77, forms a flexible hinge that is only partially detected by X-ray crystallography for hSR (Smith et al., 2010; Takahara et al., 2018). In yeast *Schizosaccharomyces pombe* SR (SpSR), this region is folded to form a short α-helix (Goto et al., 2009; Yamauchi et al., 2009), while in rat SR (rSR) it forms a loop (Smith et al., 2010). In the small domain of hSR, three α-helices surround the four β-strands (S3–S6) of the β-sheet. Two of these helices (H4 and H5) are on the same side with respect to the β-sheet and lie toward the interface with the large domain. The third helix (H6) is on the opposite site, forming a solvent-exposed surface. The large domain is formed by six β-strands, forming a twisted β-sheet (S1, S2, S7–S10) and 11 flanking α-helices (H1–H3, H7–H14) (Figures 1A,B).

**Table 1:** Structures of serine racemase available in the PDB.

**PDB code**	**Species**	**Ligands**	**Length from FASTA**	**Missing a.a**.	**Mutations**	**Open or closed conformation**	**References**
5X2L	*Homo sapiens*	PLP, Mg^2+^	348	1,2, 67–76, 318–348	–	Open	Takahara et al., 2018
3L6B	*Homo sapiens*	Malonate, PLP, Mn^2+^	348	1,2, 69–73, 330–348	C2D, C6D	Closed	Smith et al., 2010
3L6R	*Homo sapiens*	Malonate, PLP, Mn^2+^	348	1,2, 69–75, 330–348	C2D, C6D. selenomethionine labeled	Closed	Smith et al., 2010
3HMK	*Rattus norvegicus*	PLP, Mn^2+^	339	1,2, 324–339	C2D, C6D	Open	Smith et al., 2010
3L6C	*Rattus norvegicus*	Malonate, PLP, Mn^2+^	339	1, 324–339	C2D, C6D	Closed	Smith et al., 2010
1V71	*Schizosaccharomyces pombe*	PLP, Mg^2+^	323	1–5	–	Open	Goto et al., 2009
2ZR8	*Schizosaccharomyces pombe*	Serine, PDD, Mg^2+^	323	1–4	–	Closed	Goto et al., 2009
1WTC	*Schizosaccharomyces pombe*	PLP, Mg^2+^, Mg·AMP–PCP	323	1–5	–	Open	Goto et al., 2009
2ZPU	*Schizosaccharomyces pombe*	PDD, Mg^2+^	323	1–4	–	Closed	Yamauchi et al., 2009
5CVC	*Zea mays*	PLP, Mg^2+^	346	1–15, 345–346	–	Open	Zou et al., 2016

*The length of 3L6B on the PDB file is reported to be 346 a.a., but the FASTA sequence contains 348 a.a. Hence, the length of the FASTA sequence is shown in the table. Missing amino acids are those not present in the X-ray electron density*.

**Figure 2:**
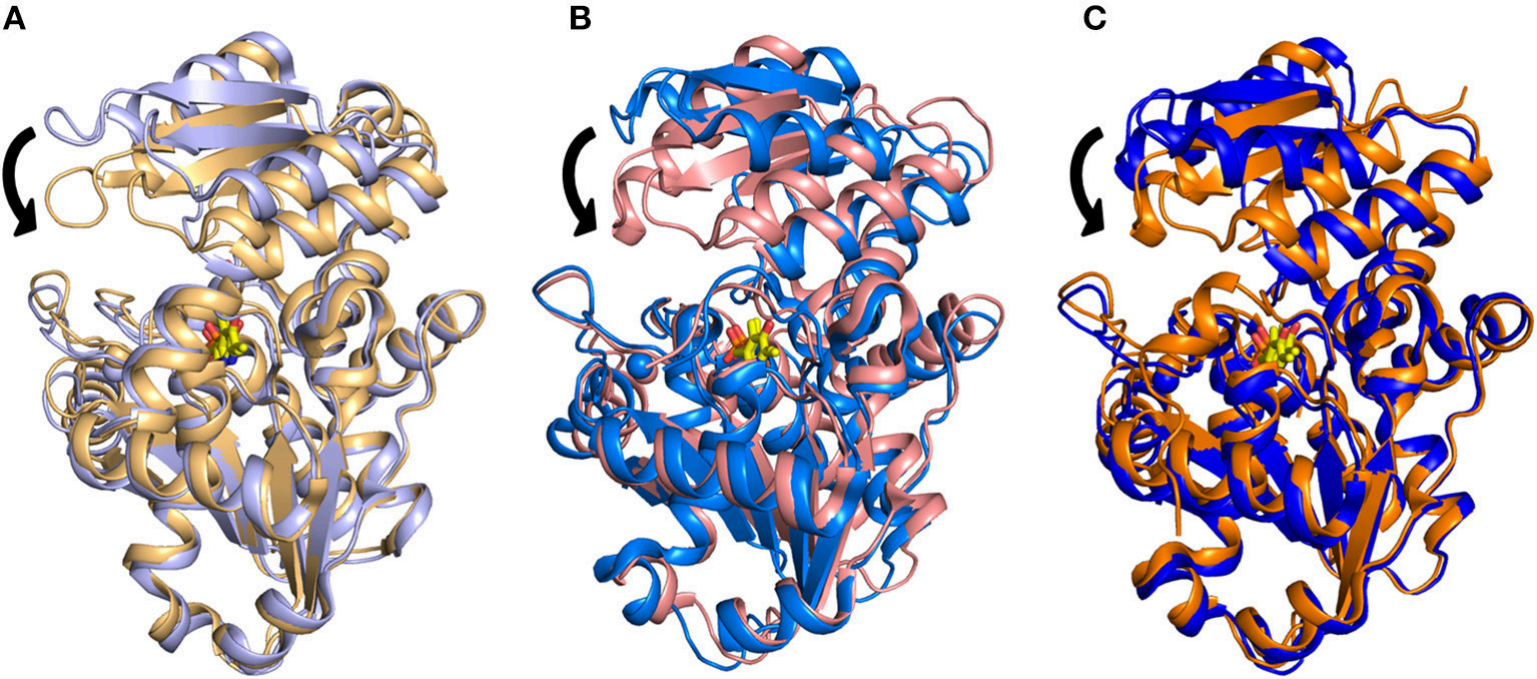
Overlay of **(A)** the open (PDB 1V71, light purple) and closed (PDB 2ZR8, light orange) structures of SpSR; **(B)** the open (PDB 3HMK, light blue) and closed (PDB 3L6C, light pink) structures for rat SR; **(C)** the open (PDB 5X2L, blue) and closed (PDB 3L6B, orange) structures of hSR. PLP is in yellow sticks. The divalent cation is reported as a sphere with the same color code of the cartoon representation. The major conformational change occurring upon ligand binding, i.e., a 20° hinge movement of the small domain toward the large domain, is indicated by arrows.

The structural investigation of SpSR (Goto et al., 2009; Yamauchi et al., 2009), which exhibits 35.1% sequence identity with hSR, allowed the detection of an open-closed conformational shift occurring upon binding of the substrate. This mechanism was described previously for fold type I PLP-dependent aspartate aminotransferases (Jansonius and Vincent, 1987; Jäger et al., 1994; Okamoto et al., 1994) and for several fold-type II enzymes such as OASS and tryptophan synthase (Raboni et al., 2009; Mozzarelli et al., 2011). The structure of SpSR without any ligand at the active site (PDB code: 1V71), is in an open conformation (Goto et al., 2009), whereas SpSR modified at the active site with a lysino-D-alanyl group—which mimics the substrate—was found to be in a closed conformation (PDB code: 2ZPU) (Yamauchi et al., 2009). Co-crystallization of this modified form with serine, which could still be accommodated at the active site despite the modification, also stabilized a closed conformation (PDB code: 2ZR8) (Goto et al., 2009). In the observed closed conformation, the small domain undergoes a 20° rotation toward the large domain to close the active site (Figure 2A). A large conformational change occurs to the asparagine loop Ser-Ser-Gly-Asn (residues 81–84 for SpSR, 83–86 for rat and human SR), at the N-terminal part of α-helix H5 (H4 in rat and human SR, since in SpSR an extra helix is present after helix H3), which forms the binding site for the carboxylate moiety of the substrate serine. Moreover, the carboxylate is involved in a salt bridge with the N-terminal Arg133 of α-helix H7 (H6 in rat and human SR) (Goto et al., 2009). An analogous open-closed conformational change was described for rSR by Smith and coworkers (Smith et al., 2010). The structure of rSR converts from an open conformation (PDB code: 3HMK) of the enzyme to a closed conformation upon formation of a complex with the competitive inhibitor malonate (PDB code: 3L6C). Arg135 (corresponding to Arg133 in SpSR) and the asparagine loop move toward the ligand bound to the active site, similarly to the behavior observed for SpSR (Figure 2B). The structure of hSR bound to malonate was also reported (PDB code: 3L6B) (Smith et al., 2010). Human and rat SR are 90% identical in sequence and their structures are almost indistinguishable (Smith et al., 2010). A comparison of the structure of hSR bound to malonate with a recently published structure of hSR in the free form (PDB code: 5X2L) (Takahara et al., 2018) confirmed that also hSR undergoes an open-closed conformational change when a ligand is present at the active site (Figure 2C). The structure of a plant SR from maize was also solved (PDB code: 5CVC), showing a fold similar to the other SR structures, apart from the C-terminal helix, which protrudes outside the core of the monomer (Zou et al., 2016).

**Figure 3:**
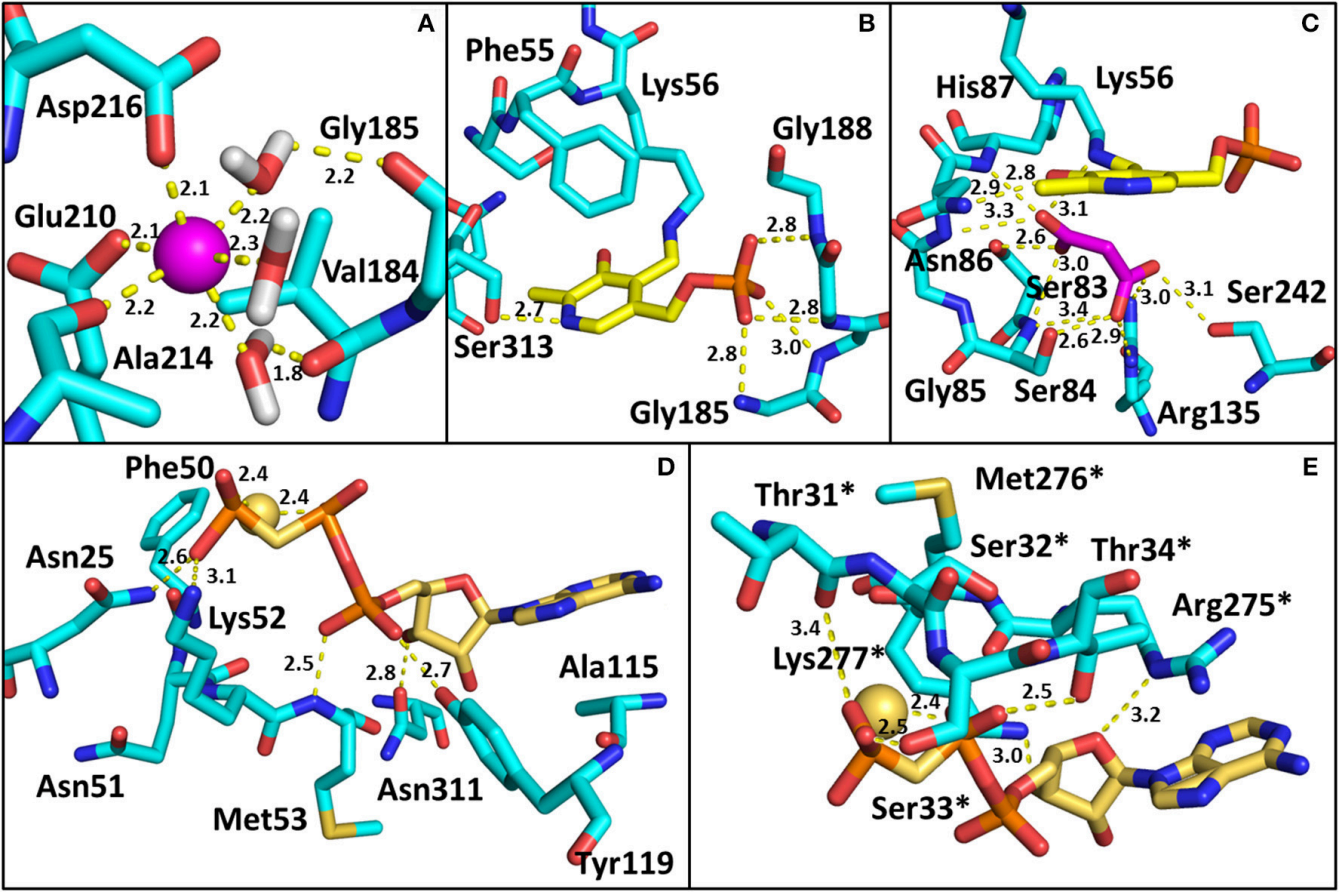
Binding sites in SR. The amino acids involved in the interactions are reported as cyan sticks, and polar interactions are highlighted by yellow dotted lines. The PDB used are 3L6B (hSR, closed form) for panels **(A–C)**, and 1WTC (spSR with AMP-PCP) for **(D,E)**. **(A)** Divalent cation binding site in hSR. The cation (Mn^2+^) is represented as a pink sphere; **(B)** PLP binding site in hSR; **(C)** Malonate binding site in hSR; **(D)** AMP-PCP binding site in spSR. The residues of the monomer in closer contact with the allosteric effector are reported. The positions of Asn25, Phe50, Asn51, Lys52, Met53, Ala115, Tyr119, and Asn311 in spSR correspond to His24, Phe49, Asn50, Lys51, Thr52, Ala117, Tyr121, and Asn316 in hSR, respectively; **(E)** residues of the second monomer involved in the interaction with AMP-PCP are reported. Asterisks indicate that the residues belong to the monomer on the opposite side of AMP-PCP. The positions of Thr31, Ser32, Ser33, Thr34, Arg275, Met276, and Lys277 in spSR correspond to Thr30, Ser31, Ser32, Ile33, Arg277, Met278, and Lys279 in hSR, respectively. Water molecules involved in the binding of SR with ligands are omitted for the sake of simplicity in all panels except **(A)**. All distances are within 3.4 Å.

**Figure 4:**
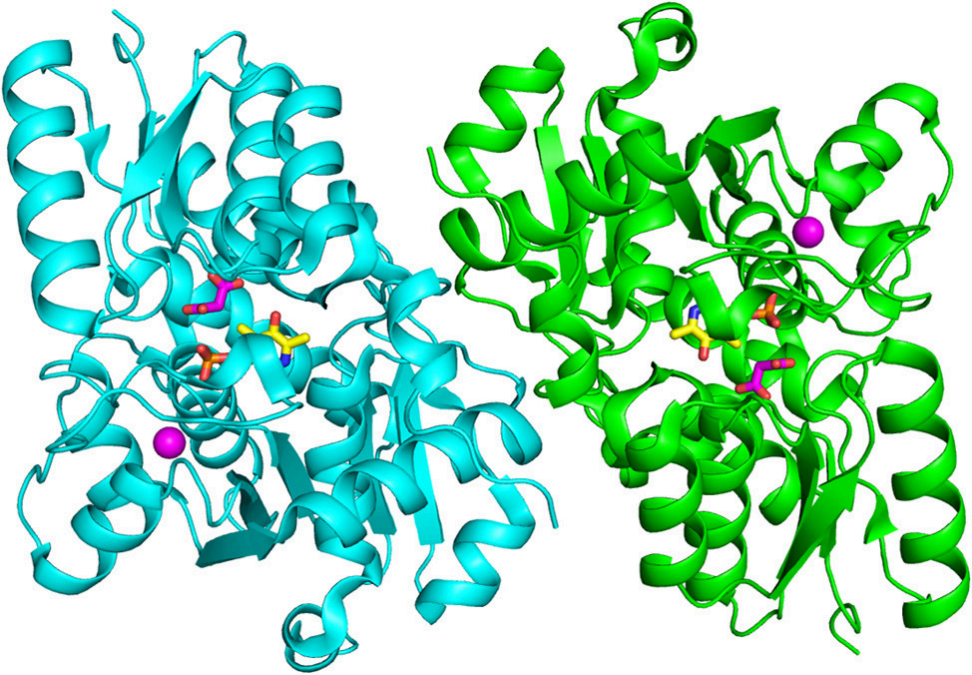
Dimeric structure of hSR (PDB code: 3L6B). The two monomers are represented in cyan and green. PLP and malonate are in sticks, and colored in yellow and pink, respectively. The divalent cation is represented as a pink sphere.

All structures of SR deposited so far in the PDB share two common features: the presence of a site for the binding of divalent cations and a similar spatial arrangement of the PLP-binding site. The binding site for divalent cations is in the large domain and is physiologically occupied by Mg^2+^. Binding of divalent cations is essential for the enzyme correct folding, stability and activity (see below) (Cook et al., 2002; Ito et al., 2012; Bruno et al., 2017). In the structures of rat and human SR (all but 5X2L), a Mn^2+^ ion is present instead of Mg^2+^, since MnCl_2_ was used in the crystallization buffer. This ion has no physiological relevance and the presence of Mn^2+^ does not alter the structure of the enzyme. The metal binding site is formed by ionic interactions with the carboxylate groups of Glu208 and Asp214 (Glu210 and Asp216 in rSR and hSR), the backbone carbonyl group of Gly212 (Ala214 in rSR and hSR) and three water molecules (Goto et al., 2009; Smith et al., 2010; Figure 3A). The metal ion is coordinated with an octahedral geometry. This site is not directly involved in catalysis, although it is connected through water molecules to a tetra-glycine loop (Gly 183-184-185-186 for SpSR and 185-186-187-188 for rSR and hSR) at the N-terminal of α-helix H9 (in SpSR numbering, H8 in rSR and hSR), which forms a series of H-bonds with the phosphate group of PLP, contributing to the correct positioning of the cofactor (Figure 3B). The PLP ring is covalently linked as an internal aldimine to a Lys residue in the active site (Lys57 in SpSR and Lys56 in rSR and hSR). PLP binds with the *re* face toward the solvent, in the same orientation as in aspartate aminotransferases (Goto et al., 2009). Considering hSR numbering, conserved residues in the PLP active site are motifs formed by residues 54–59 (Ser-X-Lys-Ile-Arg-Gly), 313–316 (Ser-X-Gly-Asn) and the tetra-glycine loop (Smith et al., 2010). Ser84 (hSR numbering), a highly conserved residue, was proved to be essential for racemase and D-serine dehydratase activities because it is involved in the binding of ligands to the active site (see below) (Yoshimura and Goto, 2008; Goto et al., 2009; Smith et al., 2010; Figure 3C). SR is present in solution as a symmetric dimer, as confirmed by X-ray crystallography, size-exclusion chromatography and glutaraldehyde cross-linking (Goto et al., 2009; Smith et al., 2010; Figure 4). Most residues at the dimer interface are conserved among different species (Goto et al., 2009). The dimer was found in both open and closed conformations. The analysis of the buried monomer-monomer surface area for rSR in the open and closed form indicated that the dimer interface has a high degree of flexibility (Smith et al., 2010), probably corresponding to a rearrangement of the interactions between the two monomers upon ligand binding to the active site, as a consequence of the open-closed conformational switch. An equilibrium between dimer and tetramer has been described (Wang and Barger, 2011), and found to depend on the presence of ligands and metal ions (Bruno et al., 2017).

**Figure 5:**
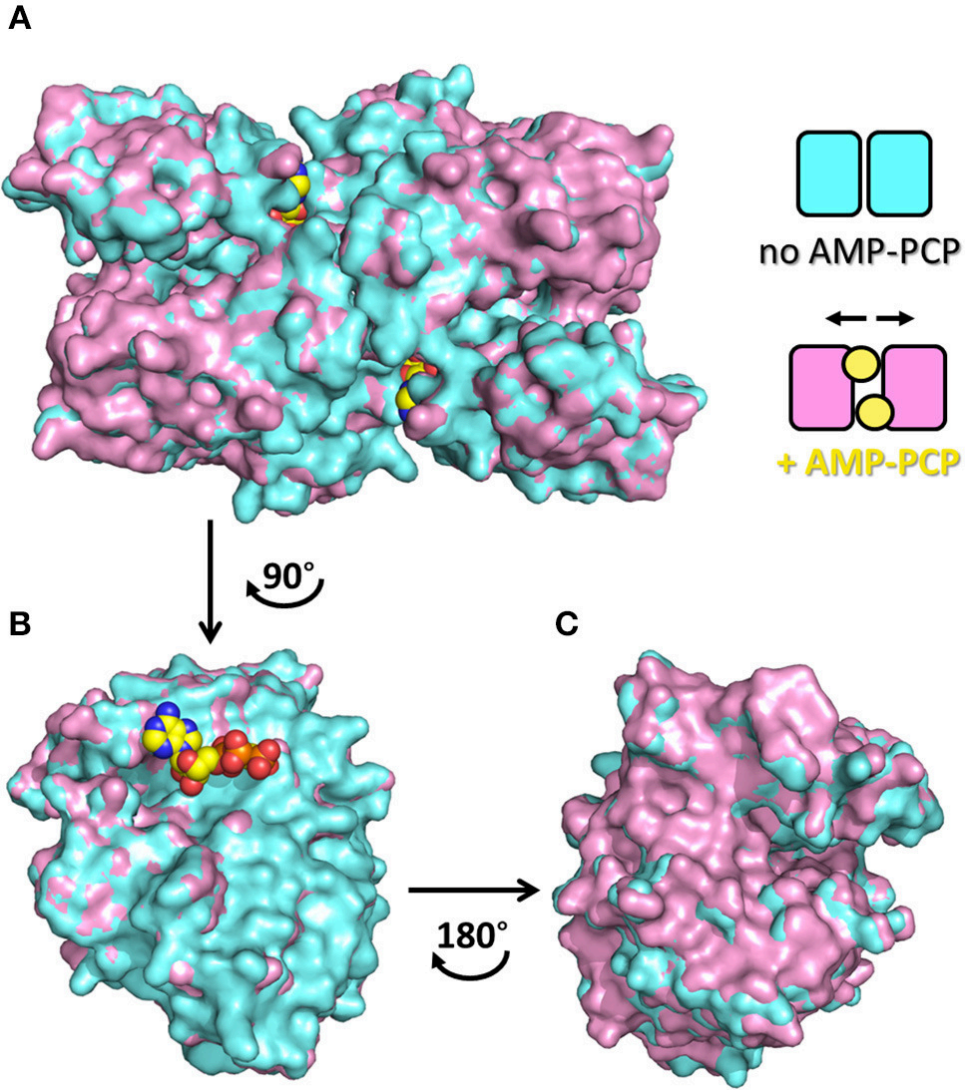
Quaternary rearrangement of spSR upon binding of AMP-PCP with enlargement of the groove between the two monomers. **(A)** Surface representation of spSR in the open form in the presence (PDB 1WTC, pink) and absence (PDB 1V71, cyan) of AMP-PCP bound to two symmetric sites at the dimer interface. The scheme indicates the changes in the relative position of the monomers in the two conditions, i.e., without or with AMP-PCP. In order to highlight the quaternary changes caused by AMP-PCP, the superposition of only one monomer is shown **(B)** facing the direction of the dimer interface, upon rotation of 90° of the dimer, and **(C)** from the opposite side of the subunit, upon rotation of 180° of the monomer, i.e. rotation of the dimer of 90° in the opposite direction. AMC-PCP is in yellow. In **(B)**, only the molecule of AMP-PCP bound to the monomer is shown.

The dimeric structure of SR is crucial for the regulation of enzyme activity. The structure of SR bound to a stable analog of ATP, 5′-adenylyl methylene diphosphonate (AMP-PCP), in the absence of ligands bound to the active site, i.e., in the open form, was solved for SpSR (Goto et al., 2009). AMP-PCP in complex with Mg^2+^ ions binds into a cleft at the interface between the subunits at two symmetry-related sites. AMP-PCP interacts with the small and large domains of one subunit, and with the large domain of the other subunit. The binding of Mg·AMP-PCP to the open form changes the relative orientation between the two subunits, increasing the width of the groove between the two monomers (Figure 5). The residues involved in AMP-PCP binding include Ala115 (SpSR numbering) and Tyr119, interacting within the small domain. The large domain binds AMP-PCP with Asn25, Phe50, Asn51, Lys52, Met53, and Asn311, all residues present in loop regions (Figure 3D). On the opposite side, the ligand interacts with Thr31, Ser32, Ser33, Thr34, and Arg275, Met276, Lys277, both at a terminal part of α-helices of the large domain of the second monomer in the dimer (Figure 3E). Water molecules are also involved in the binding. The position of Mg·AMP-PCP has been docked into the structure of hSR in the presence of malonate (PDB code: 3L6B), suggesting that the overall interaction with ATP is similar between yeast and mammalian SR (Jirásková-Vanícková et al., 2011). Although the AMP-PCP site and the substrate binding site are 15 Å away, and the two symmetric ATP sites are 24 Å apart, an allosteric communication occurs, likely by rearrangement of a H-bond network, which connects the O3′ group of PLP with the γ-phosphate of AMP-PCP. This network involves Thr52, Asn86, Gln89, Glu283 and Asn316 in hSR (Met53, Gln87, Glu281, Asn311 in SpSR) and two water molecules (Goto et al., 2009; Marchetti et al., 2013; Canosa et al., 2018).

## SR Catalysis

SR catalyzes two reactions, the reversible racemization of L-Ser or D-serine and the irreversible dehydration of L-Ser and D-Ser to pyruvate and ammonia (De Miranda et al., 2002; Foltyn et al., 2005).

**Scheme 1:**
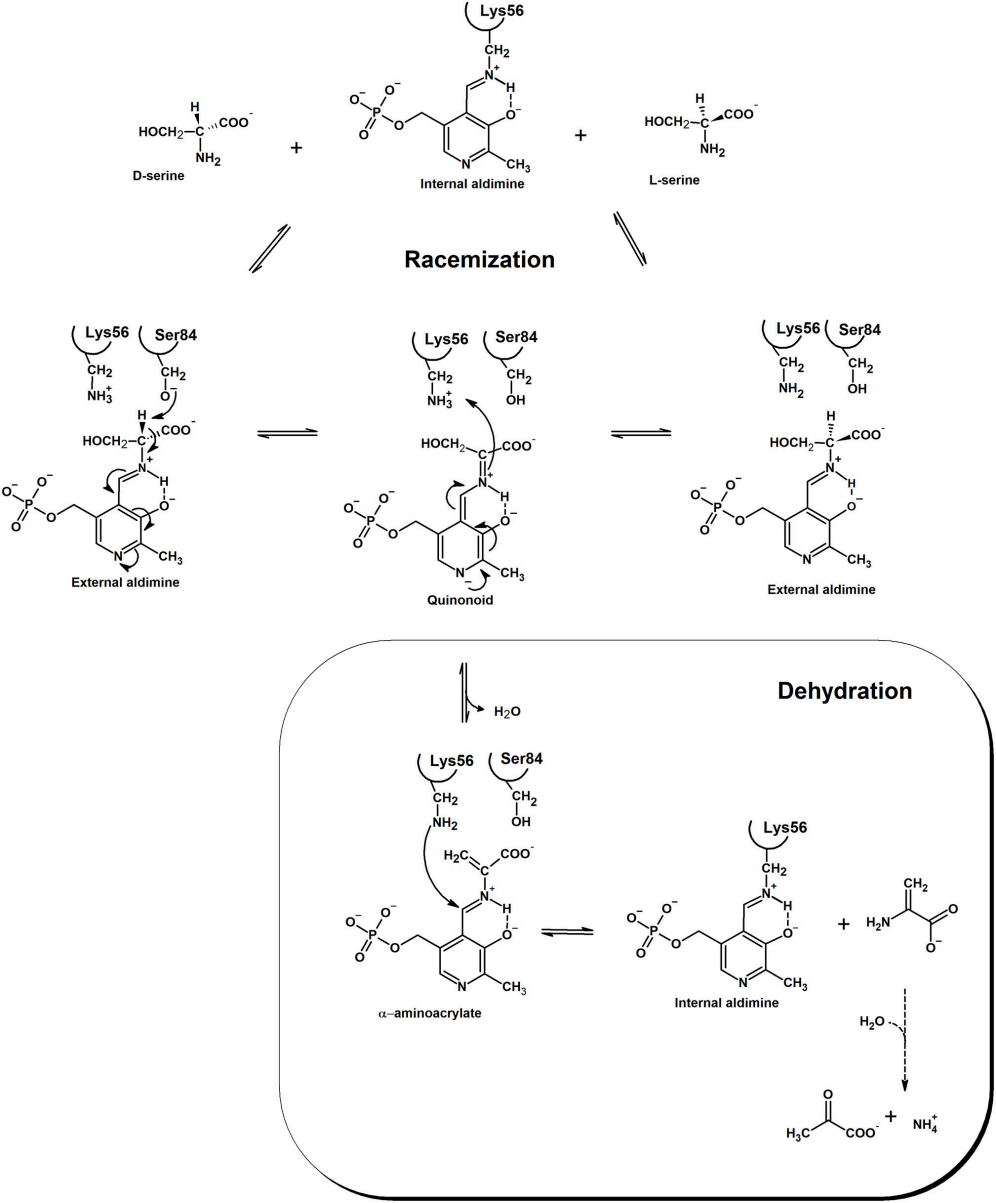
Mechanism of the racemization and dehydration reactions catalyzed by SR. Electron movement is shown in the direction of D-Ser racemization. The product of the dehydration reaction, α-aminoacrylate, is spontaneously and rapidly hydrolyzed to pyruvate and ammonia. Residue numbering refers to the human ortholog. Modified from Goto et al. (2009) and Smith et al. (2010).

Racemases have been classified as PLP-dependent or PLP-independent (Conti et al., 2011), with SR belonging to the first group. Amino acid racemization is a two-steps reaction where alpha proton abstraction is followed by re-protonation from the opposite side of a planar intermediate. Since the pK_a_ of alpha proton is considerably high, pK about 21 or higher (Yoshimura and Esak, 2003), racemases have evolved to face the problem of increasing the acidity of this proton. In hSR, PLP is covalently bound to Lys56 and, differently to fold type I PLP-dependent enzymes (Griswold and Toney, 2011), presents a deprotonated pyridine nitrogen (Goto et al., 2009). This feature is shared with fold type II PLP-dependent enzymes, like bacterial alanine racemase (Toney, 2005), tryptophan synthase (Raboni et al., 2009) and *O*-acetylserine sulfhydrylase (Mozzarelli et al., 2011). Lys56 is not only involved in the formation of the internal aldimine with PLP, but, together with Ser84, is also a catalytic residue and participates in proton abstraction/reprotonation of serine in a classical two-base mechanism (Foltyn et al., 2005; Goto et al., 2009; Scheme 1). In the deprotonated form, the substrate binds to SR active site and undergoes a transaldimination reaction with PLP with formation of an external aldimine. The protonation state of Ser84 and Lys56 depends on the enantiomer of serine that binds to the enzyme. When L-Ser is the substrate, a deprotonated Lys56 can abstract the alpha proton leading to an anionic quinonoid intermediate. When D-Ser binds, Lys56 is protonated and Ser84 is deprotonated, thus allowing proton abstraction from the opposite side of the amino acid. The existence of a true quinonoid species is questioned by the lack of any experimental observation of this intermediate and by the structural evidence that a negative charge on the pyridine nitrogen could not be stabilized by a positively charged residue, as observed for transaminases (Griswold and Toney, 2011). On the other hand, the formation of a metastable quinonoid would favor reaction specificity, as already observed for alanine racemase (Toney, 2005). The final step of the reaction is reprotonation by either Ser84 or Lys56. Human SR does not racemize L-Thr, whereas archaeal SR does, albeit with lower efficiency with respect to serine (Ohnishi et al., 2008). However, very recent works have demonstrated that mouse SR (mSR) can catalyze the racemization of L-Asp (Ito et al., 2016). The efficiency of D-Asp production is more than 500-fold lower than that of D-Ser production (k_cat_/K_m_ = 12 min^−1^·mM for L-Ser racemization vs. 0.022 min^−1^·mM for L-Asp racemization). Over-expression of SR in cultivated cells increases the concentration of D-Asp, thus suggesting another relevant role *in vivo* for this enzyme. Phylogenetic analyses indicated that animal Asp and Ser racemases form a serine/aspartate racemase family cluster (Uda et al., 2016) and the aspartate racemase activity evolved from the SR activity by acquisition of a triple serine loop facing the substrate binding site (Uda et al., 2016, 2017).

The anionic intermediate that forms upon alpha proton abstraction can undergo a β-elimination reaction with formation of the α-aminoacrylate, an unstable intermediate that is readily hydrolyzed to pyruvate and ammonia with restoration of the internal aldimine. The β-elimination reaction is also catalyzed on β-chloroalanine, L-threonine, L-Ser-*O*-sulfate and L-threo-3-hydroxyaspartate (Panizzutti et al., 2001; Strísovský et al., 2005) and does not lead to syncatalytic inactivation of the human enzyme, an event often observed in transaminases (Morino et al., 1979; Cooper et al., 2002). Both racemization and dehydration reactions are activated, to a different extent, by ATP (see below), the dehydration of L-Ser being the most affected, with a 31-fold increase in catalytic efficiency upon nucleotide binding (De Miranda et al., 2002; Canosa et al., 2018; Table 2). Under physiological conditions, i.e., SR fully saturated with ATP, the dehydration reaction is several folds more efficient than the racemization reaction. The physiological relevance of D-Ser production by SR has been demonstrated with knock-out mice, which show a D-Ser concentration in the brain that is less than 10% compared to normal mice (Labrie et al., 2009; Balu et al., 2013). On the other hand, the role of dehydration *in vivo* is still debated and was suggested to be relevant for D-Ser degradation, especially in those brain areas lacking D-amino acid oxidase (De Miranda et al., 2002). Since SR has evolved from serine dehydratases (De Miranda et al., 2000), it is likely that the dehydration reaction is the vestige of the enzyme ancestor and was maintained during evolution for its contribution to D-Ser homeostasis. Overall, the efficiency of SR in D-Ser production is very low, likely due to the low metabolic requirement for this amino acid and the need for a strict regulation of its production. However, there is evidence that localization and/or interaction with other proteins might play a role in increasing the rate of D-Ser production under physiological conditions (see below).

**Table 2:** Catalytic parameters of hSR for serine racemization and dehydration in the presence and absence of ATP, at saturating concentrations of Mg^2+^.

**Reaction**	**K**_****M****_ **(mM)**		**k**_****cat****_ **(min**^****−1****^**)**		**k_cat_/K_M_ (s^−1^·M^−1^)**		**Fold increase**
	**–ATP**	**+ATP**	**–ATP**	**+ATP**	**–ATP**	**+ATP**	
L-Ser racemization	35 ± 5	34 ± 4	19 ± 1	35 v1	9.2 ± 1.4	17.5 ± 2.1	1.9
L-Ser dehydration	76 ± 10	12 ± 1	37 ± 4	183 ± 3	8.1 ± 1.1	253.0 ± 15.0	31.0
D-Ser dehydration	46 ± 3	167 ± 16	1.7 ± 0.1	23 ± 1	0.6 ± 0.1	2.4 ± 0.1	4.0

*Data are from Marchetti et al. (2013) and Canosa et al. (2018)*.

## SR Small Ligands and Protein Interactors

hSR activity is finely regulated by several physiological effectors, including ATP, cations, anions and interacting proteins, whose fluctuations contribute to the homeostasis of D-serine.

### Small Ligands

The stimulation of SR activity by ATP was first described by Neidle and Dunlop (Neidle and Dunlop, 2002) on the murine ortholog, when they observed a five-fold increase in the catalytic activity in the presence of small quantities of yeast extract. Successive experiments demonstrated that SR activity was triggered by either magnesium or calcium and nucleotides, the most active being ATP, ADP and GTP. In the same year, other two research groups independently confirmed the activation role of divalent cations and nucleotides on mSR activity (Cook et al., 2002; De Miranda et al., 2002). The effectiveness of ADP and non-hydrolyzable ATP analogs (De Miranda et al., 2002; Neidle and Dunlop, 2002) further demonstrated that the nucleotides do not provide an energetic contribution to catalysis.

ATP is one of the principal modulators of hSR catalysis and takes part in a fine-tuning of the enzymatic activity (Table 2). The catalytic efficiency of dehydration on L-serine is deeply enhanced by ATP, which exerts a 31-fold increase in activation, with a k_cat_/K_M_ of 8.1 ± 1.1 and 253.0 ± 15.0 s^−1^ M^−1^ in the absence and presence of ATP, respectively (Marchetti et al., 2013). Otherwise, under the same conditions, the efficiency of dehydration activity on D-serine increases 4-fold, with only a small effect of ATP on D-serine degradation rate (0.6 and 2.4 s^−1^ M^−1^, respectively). The racemization of L-serine occurs with about a two-fold increased efficiency in the presence of ATP (from 9.2 to 17.5 s^−1^ M^−1^), but the net effect of ATP binding is a strong stimulation of L-serine degradation, with an increment from 0.9 to 14.5 of the ratio between the two activities in the absence and presence of ATP, respectively (Marchetti et al., 2013). As shown above, the comparison of the structure of SpSR in the absence and presence of the ATP analog AMP-PCP (PDB 1V71 and 1WTC) revealed a change in the relative orientation of the major and minor domain (Goto et al., 2009), resulting in a wider back groove. Moreover, in the presence of ATP and either glycine or malonate, hSR active site appears less accessible than in the absence of ATP, as demonstrated by PLP fluorescence quenching (Marchetti et al., 2015). Moreover, by monitoring PLP fluorescence in the absence and presence of ATP, it was demonstrated that ATP binding to hSR promotes a decrease in the polarity of the active site, diminishing its accessibility (Marchetti et al., 2013). These observations are in line with the existence of a H-bond network that links the ATP binding site with the active site (Goto et al., 2009) and modulates the active site open-to-closed transition, promoting the correct orientation of the catalytic residues. Recently, Gln89 has been pointed out as a key residue in the allosteric communication between the active site and the ATP-binding site. This residue is specifically involved in the activation of the dehydration reaction by ATP and its mutation to either Ala or Met completely abolishes nucleotide-dependent modulation of serine dehydration (Canosa et al., 2018).

Based on the intracellular ATP concentration, hSR was thought to be always saturated *in vivo*. However, *in vitro* studies revealed that ATP binds with a strong cooperativity (Hill n close to 2) with calculated ATP K_D_s for high and low affinity states of 11.5 μM and 1.8 mM, respectively. These values fall within the physiological range (Marchetti et al., 2013), underlying a potential sensitivity of hSR activity on intracellular ATP fluctuations. The apparent K_D_s for ATP obtained through fluorescence measurements in the absence of active site ligands (0.26 ± 0.02 mM) and through activity assays (0.22 ± 0.01 mM and 0.41 ± 0.02 mM for L-serine and D-serine dehydration, 0.22 ± 0.05 mM for L-serine racemization, respectively), are very similar. This finding indicates that the intermediates that form during the catalytic cycle do not allosterically affect ATP binding site, in contrast with glycine that forms a stable Schiff base and affects ATP affinity (Dunlop and Neidle, 2005; Marchetti et al., 2013). Furthermore, when the active site is involved in the formation of a stable complex between PLP and a ligand, such as glycine, ATP binds to hSR in a non-cooperative fashion, with a 50-fold stronger affinity. In the same way, ATP influences the substrates affinity for the active site (as mirrored on K_M_ values) and strengthens the binding of covalent (i.e., glycine, 15-fold) or non-covalent (i.e., malonate, 10-fold) inhibitors (Marchetti et al., 2013, 2015). Based on these evidences, glycine, the alternative glutamate co-agonist to D-serine on NMDA receptors, may participate to the fine-tuning of the communication between active and allosteric sites.

As for ATP, also the residues involved in the metal binding site are highly conserved in the yeast, murine and human SR sequences, giving a similar coordination in all the three structures (see above) (Goto et al., 2009; Smith et al., 2010; Takahara et al., 2018). Although the mammalian enzyme can be activated both by calcium or magnesium (Cook et al., 2002; De Miranda et al., 2002; Neidle and Dunlop, 2002), the metal binding site has always been assumed as physiologically occupied by the latter because of its intracellular concentration and the stronger activation effect with respect to the former. Recently, investigations of hSR enzymatic activity *in vitro* in the presence of either magnesium or calcium support the absence of a relevant competition between the two ions at physiological level (Bruno et al., 2017). Racemization and dehydration activities in the presence of 1 mM L-serine and 2 mM ATP, concentrations close to intracellular conditions in neurons (Gribble et al., 2000; Foltyn et al., 2005; Genc et al., 2011), unveiled a larger effect of magnesium on catalytic efficiency, with a 2.5-fold difference with respect to calcium. Magnesium and calcium bind to hSR with similar affinities both in the absence (EC_50_ of 28 ± 3 μM and 126 ± 7 μM, respectively) and presence of ATP (EC_50_ of 17 ± 1 μM and 194 ± 6 μM, respectively). Therefore, the two cations are able to elicit with different affinities similar conformational changes responsible for the activation of hSR, thus behaving as activators (Purich and Allison, 2002). During the neural transmission, Ca^2+^ concentration locally rises up to 100 μM in neurons, but cannot compete with magnesium for the binding to hSR and does not influence the enzymatic turnover number (Bruno et al., 2017). For these reasons, the impact on hSR regulation *in vivo* is likely to be insignificant.

It has been demonstrated that the activity of hSR can be further affected by halides (Marchetti et al., 2015). Among them, chloride, the only one with physiological relevance, behaves like an “uncompetitive activator” (Wild et al., 1976; Maruyama, 1990), since it influences both k_cat_ and K_M_, but does not alter the catalytic efficiency (Marchetti et al., 2015). In mature neurons in the CNS, the intracellular concentration of chloride is maintained at about 5 mM through the co-transport of different ionic species (Doyon et al., 2016). During the action potential, the intracellular concentration of Cl^−^ can increase five-fold, to 20–25 mM, both in the presynaptic terminal and in the postsynaptic dendrites. This rapid change occurs due to the activation of GABA_A_ and glycine receptors, and modulates both the release of glutamate neurotransmitter and the inhibitory post-synaptic current (Price and Trussell, 2006). It is interesting to note that *in vitro* studies show that in the presence of ATP and Mg^2+^, hSR quaternary structure can be affected by the concentration of NaCl. In particular, hSR exists as a tetramer in the absence of NaCl, whereas at increasing salt concentration the equilibrium is shifted toward the dimeric form (Bruno et al., 2017). The half effect corresponds to 20–50 mM NaCl, suggesting a possible role of chloride in the oligomeric distribution of hSR.

### Protein Interactors

In addition to small molecule effectors and post-translational modifications (see below), SR function is regulated by the interaction with specific proteins, particularly proteins associated with AMPA and NMDA receptors. Particularly, the C-terminal end, located near the ATP binding site, mediates the interaction with protein partners.

The Glutamate Receptor Interacting Protein (GRIP) enhances SR activity and D-serine release from glia (Kim et al., 2005) and in transfected mammalian cells (Baumgart et al., 2007). Furthermore, GRIP was shown to cause SR conformational changes (Baumgart et al., 2007). This conclusion was supported by molecular modeling of the interaction between SpSR and a GRIP-contained PDZ domain (Baumgart et al., 2007).

Protein Interacting with C-kinase (PICK1) is activated by the erythropoietin-producing hepatocellular receptor (Eph), which promotes its release in the cytosol of astrocytes, where it interacts with SR. Specifically, upon Eph receptor activation, there is a dissociation of PICK1 from Eph and an increased association with SR (Zhuang et al., 2010; Kiriyama and Nochi, 2016), accompanied with an increase in D-serine synthesis (Fujii et al., 2005; Hanley, 2008; Hikida et al., 2008). GRIP and PICK1 both contain a PDZ domain, which is recognized by three carboxyl-terminal amino acids of SR with a well characterized consensus sequence (Val-Ser-Val) (Baumgart et al., 2007). How PICK1 and GRIP interact to regulate SR is still unclear, but they are both dependent on the phosphorylation status of AMPA receptors (Wolosker et al., 1999; Fujii et al., 2005; Kim et al., 2005). Phosphorylation at Ser880 causes dissociation of GRIP, whereas PICK1 remains bound (Mustafa et al., 2004; Wang and Barger, 2011). Other accessory proteins, such as Stargazin and postsynaptic density proteins 95 (PSD-95), also regulate AMPA receptors (Ma et al., 2014). There is evidence of the formation of a ternary complex of SR with Stargazin and PSD-95, presumably affecting SR activity and, therefore, glutamate neurotransmission (Ma et al., 2013).

Another interactor of SR is Golga-3, a protein that binds to the cytosolic face of the Golgi apparatus and stabilizes SR levels through inhibition of its ubiquitination (Wolosker et al., 1999; Fujii et al., 2005; Dumin et al., 2006; Canu et al., 2014). Recently, it was also showed that the protein Disrupted in Schizophrenia 1 (DISC1), which is implicated in pathology of major psychiatric disorders, binds SR, preventing its ubiquitination and degradation. The C-terminus truncated form of DISC1 is unable to bind SR and induces its degradation and D-serine depletion (Ma et al., 2013). FBXO22, a component of the ubiquitin-proteasome system, also interacts with SR modifying its intracellular organization (see below) (Dikopoltsev et al., 2014).

## SR Post-Translational Modifications

SR was reported to be post-translationally modified by nitrosylation, phosphorylation and palmitoylation. SR levels are also regulated by ubiquitin-tagging for proteasomal degradation.

### Nitrosylation

**Figure 6:**
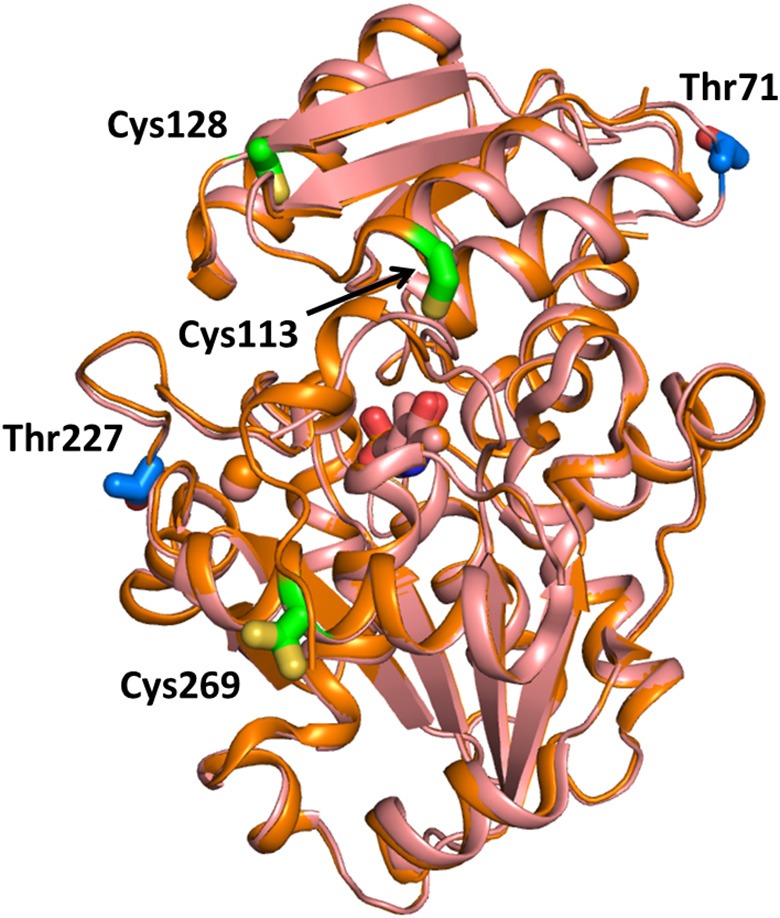
Sites for SR nitrosylation and phosphorylation. The structure of hSR (PDB 3L6B, light pink) and rSR (PDB 3L6C, orange) are superimposed and the positions of the sites for S-nitrosylation (in green on the structure of hSR) and phosphorylation (in blue on the structure of rSR) are reported in sticks. The divalent cation is in pink. Cys269 has two possible positions with 0.5 occupancy based on the PDB file.

Experiments on a human glioblastoma cell line showed that the activity of SR was inversely regulated by nitric oxide, and addition of D-serine promoted denitrosylation of the murine purified ortholog (Shoji et al., 2006). These results suggested a regulation mechanism in which D-serine, besides being the substrate of the enzyme, is also an activator through denitrosylation (Shoji et al., 2006). The nitrosylation site in murine SR was determined to be Cys113, adjacent to the ATP binding site, whose S-nitrosylation was shown to inhibit the enzyme activity of about 10-fold (Mustafa et al., 2007). This regulation mechanism was interpreted as a feedback control of NMDA activation (Mustafa et al., 2007). Unlike the murine ortholog, human SR was shown to be S-nitrosylated at three cysteine residues, Cys113, Cys269, and Cys128, with Cys269 being unique to the human ortholog (Marchesani et al., 2018; Figure 6). The inhibition kinetics was biphasic, indicating that at least two of these residues were responsible for enzyme inhibition, as confirmed by site-directed mutagenesis of Cys113 (Marchesani et al., 2018). When nitrosylated, hSR binds ATP with affinity and cooperativity similar to the native enzyme. However, nitrosylation does not affect enzyme activity in the absence of ATP, suggesting that nitrosylation alters the allosteric communication between the ATP binding site and the active site, possibly via the stabilization of a distinct conformation (Marchesani et al., 2018).

### Phosphorylation

Phosphorylation of murine SR takes place at Thr71 in the cytosolic and in the membrane-bound SR. In the latter, an additional phosphorylation site was detected at Thr227 (Balan et al., 2009; Foltyn et al., 2010; Figure 6). Phosphorylation at Thr71 is the main phosphorylation site and promotes D-serine synthesis by increasing enzyme turnover rate. Indeed, Thr71Ala SR exhibits a 50% lower activity when compared to the wild type. However, this phosphorylation site is not conserved in hSR, indicating that it belongs to a rodent-specific regulation mechanism. Phosphorylation of Thr227 favors the association of SR with membranes. Indeed, the levels of membrane-bound SR under non-stimulated conditions are reported to decrease in Thr227Ala mutant. Prediction of the kinases mediating SR phosphorylation at Thr71 and Thr227 carried out by *in silico* analysis of the phosphorylation motif identified proline-directed kinases as the strongest candidates. However, treatment with inhibitors of the predicted kinases did not alter the phosphorylation profile nor changed SR binding to the membrane making the identification of the specific kinase still elusive. In addition, phosphorylation at Thr71 was reported to be regulated by phosphatases and stimulated by growth factors present in the serum, as expected for a dynamic event. Phosphorylation by protein kinase C (PKC) on serine residue of rodent SR was suggested by Mustafa and colleagues based on the proximity of the two proteins mediated by PICK1 binding (Mustafa et al., 2004). Later, Vargas-Lopes et al. demonstrated that PKC phosphorylates SR on serine residues and decreases SR activity *in vitro* (Vargas-Lopes et al., 2011). Analogously, PKC activation increases SR phosphorylation and reduces D-serine levels in astrocyte and neuronal cultures and in rat frontal cortex. In particular, PKC-mediated phosphorylation regulates D-serine availability in the brain in NMDA-dependent memory-related processes. Alignment of the amino acid sequences of human, rat and mouse SR revealed five conserved consensus sequences for PKC phosphorylation but the site remains to be established.

### Acylation/Palmitoylation

SR does not possess specific amino acid motifs required for prenylation, isoprenylation, or myristoylation. Interestingly, SR is a rare example of an *O*-palmitoylated protein at still unidentified serine or threonine residues (Balan et al., 2009). Palmitoylation contributes to membrane binding and plays a key role in SR translocation from the cytosol. In addition to [^3^H]palmitic acid, [^3^H]octanoic acid was also incorporated into SR in neuroblastoma cells, suggesting that *in vivo O*-acylation may involve fatty acids of different lengths (Balan et al., 2009).

### Ubiquitination

SR has a relatively short half-life compatible with the existence of an efficient degradation/regulatory system. Indeed, it was demonstrated that SR undergoes poly-ubiquitination in an ATP-dependent manner both *in vitro* and *in vivo*, which leads to degradation by the ubiquitin-proteasome system. However, the E3 ubiquitin ligase that transfers ubiquitin chains to SR is still unidentified (Dumin et al., 2006). The ubiquitin system is a key regulator of SR and D-serine levels. Partner proteins have been reported to affect the rate of protein degradation by the ubiquitin-proteasome system, altering its half-life and modulating SR function (see above). In particular, interaction with Golga-3 both *in vitro* and *in vivo* stabilizes SR, prevents its ubiquitination and slows down its degradation by the ubiquitin-proteasome system. Consequently, the significant increase in its half-life and steady-state levels indirectly raises D-serine levels. Analogously, DISC1 diminishes SR ubiquitination acting as a scaffold that stabilizes SR. Truncation of the C-terminus in a mutant DISC1 disrupts the physiologic binding to SR and increases ubiquitination and degradation of SR in astrocytes with a consequent decrease in D-serine production (Ma et al., 2013). FBXO22, an F-box motif-containing protein and a component of the SCF ubiquitin ligase complex, does not promote SR ubiquitination nor its targeting to the proteasome system. Indeed, it is the free FBXO22a species, without the participation of the SCF-FBXO22a complex, which prevents the accumulation of membrane-bound SR species and regulates D-serine synthesis (Dikopoltsev et al., 2014).

## SR Inhibitors

High concentrations of D-serine in the brain are associated with high NMDA receptors activity, leading to severe excitotoxicity, as observed in Parkinson and Alzheimer diseases, amyotrophic lateral sclerosis and ischemia. Therefore, significant efforts have been directed toward the development of active-site inhibitors with potential therapeutic effects (Conti et al., 2011; Jirásková-Vanícková et al., 2011).

### Active Site Ligands

**Scheme 2:**
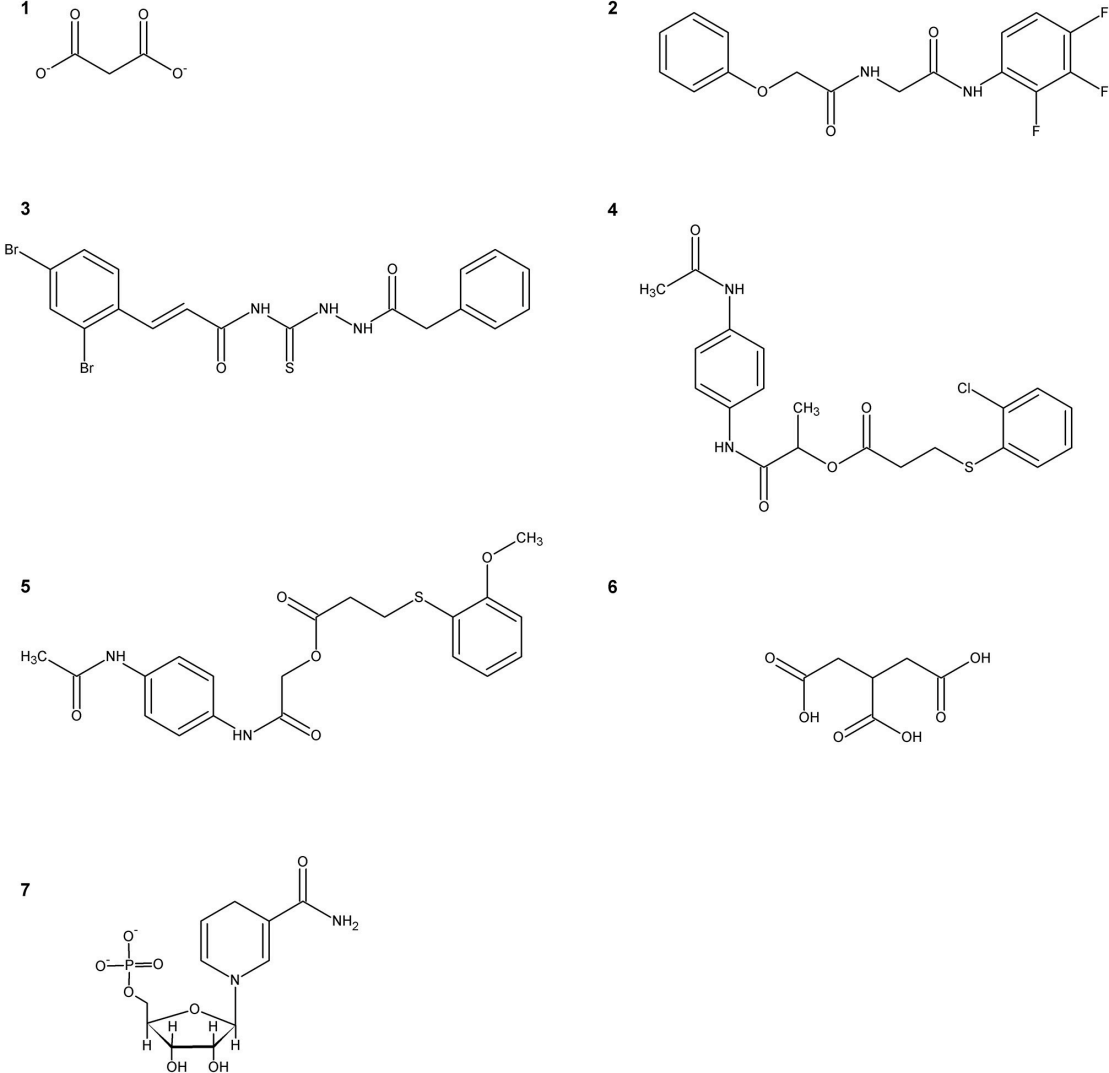
Representative inhibitors of SR.

The first SR inhibitors were identified by screening a series of dicarboxylic acids (Strísovský et al., 2005). Malonate (**compound 1**, Scheme 2) emerged as the most active compound, with a K_i_ of 27 μM for mSR, and 77 and 710 μM for hSR, in the presence and absence of ATP, respectively (Marchetti et al., 2015; Table 3). The structure of the malonate-hSR complex (Figure 3C) indicated that malonate is H-bonded to the protein backbone and to amino acid residues. Also three water molecules are involved in the binding (Goto et al., 2009; Smith et al., 2010). Attempts to improve the affinity led to 2,2-dichloromalonate, which exhibits a K_i_ of 19.3 μM for mSR (Vorlová et al., 2015). Other SR inhibitors were identified in small peptide libraries containing the 3-phenylpropanoic acid moiety (Dixon et al., 2006). Nonspecific hydroxamic acid derivatives were also identified as inhibitors of SR (Hoffman et al., 2009). However, the affinities of all these inhibitors were lower than 2,2-dichloromalonate.

**Table 3:** Selected inhibitors of hSR.

**Compound**	**K_**i**_ (μM)**	**Type of inhibition**	**Site of inhibition**	**References**
Malonate	710 ± 33 (- ATP) 77 ± 9 μM (+ATP)	Reversible	Active site	Marchetti et al., 2015
Glycine	7000 ± 300 (-ATP) 470 ± 30 (+ATP)	Reversible, covalent	Active site	Marchetti et al., 2013
**3**	^*^4	Reversible	Active site	Mori et al., 2017
**5**	^*^207	Reversible	Active site	Takahara et al., 2018
**6**	1300	Reversible	Active site	Dellafiora et al., 2015
Dicarboxylic cyclopropane	900	Reversible	Active site	Beato et al., 2016
**7**	18 ± 7	Reversible	ATP site	Bruno et al., 2016

^*^K_i_ calculated from IC50 using the equation K_i_ = IC_50_/([S]+K_M_)+1 that can be applied to reversible competitive inhibitors. Values of L-serine concentration and K_M_ were derived from data reported in the quoted references.

By a combination of *in silico* screening and *de novo* synthesis, **compound 2** was identified (Mori et al., 2014), and further optimized to generate **compound 3**, which showed an IC_50_ *in vitro* of 14 μM for mSR, a calculated K_i_ of about 4 μM (Table 3) and an *in vivo* activity on a mouse model, suppressing neuronal over-activation (Mori et al., 2017). An inhibitor with an IC_50_ of 1 mM (**compound 4**) was identified and optimized by structure-based drug design starting from hSR, leading to **compound 5**, with an IC_50_ of 0.84 mM (Takahara et al., 2018) and a calculated K_i_ of 0.207 mM (Table 3).

In order to expand the chemical space of hSR inhibitors, *in silico* structure-based screening using both the open and the closed conformations of hSR was carried out (Dellafiora et al., 2015). Compounds with very heterogeneous chemical scaffolds were identified, sharing the presence of carboxylate moieties or carboxylate isosters. They exhibit K_i_ values in the low millimolar range, such as **compound 6** that possesses three carboxylate moieties and exhibits a calculated K_i_ of 1.3 mM (Table 3).

A further effort was carried out to mimic malonate with a cyclopropane scaffold synthesizing a small library of substituted cyclopropane derivatives. These compounds were docked into hSR structures and evaluated *in vitro*. The most active compound was dicarboxylic cyclopropane, which binds with a K_i_ of 0.9 mM (Beato et al., 2016).

The goal of developing high affinity, competitive inhibitors based on analogs of the substrate L-serine was initially perceived as relatively easy, as structural models for both open and closed conformations in complex with a few ligands were available for computer-assisted drug design. However, the results obtained so far indicate that SR is a rather difficult target for drug discovery. More structures of hSR in the presence of ligands should be of help in mapping the conformational ensemble. Furthermore, the so far identified inhibitors exhibit a relatively low affinity making rather difficult to saturate the active site and thus to obtain a high-resolution structure of the enzyme-ligand complex, hampering a structure-based drug discovery approach. In addition, it should be pointed out that hSR does not easily crystallize.

### Allosteric Ligands

It was reported that the reduced form of NADH inhibits SR activity (Suzuki et al., 2015). This finding hints for a potential link of D-serine concentration to the glycolytic flux. A well-defined metabolic link between D-serine concentration and the glycolytic flux is represented by the fact that L-serine is generated in three steps from phosphoglycerate that is a main glycolytic metabolite. The structural similarity of NADH with ATP, which acts as an allosteric effector activating hSR, suggested a potential competition for the same binding site. Indeed, it was found that the affinity of ATP for hSR in the presence of NADH decreased and the binding lost cooperativity (Bruno et al., 2016). NADH analogs based on the reduced N-substituted 1,4 dihydronicotinamidic ring, including 1-methyl-1,4-dihydronicotinamide (MNA-red) and β-1,4-dihydronicotinamide monucleotide (NMN-red) (**compound 7**, Scheme 2) were found to be partial inhibitors of hSR activity. In particular, NMN-red exhibited a mixed-type inhibition, with a K_i_ of 18 ± 7 μM (Bruno et al., 2016; Table 3). By molecular docking, the binding site for NADH, MNA-red and NMN-red was identified to be at the dimer interface, close to the ATP binding site (Bruno et al., 2016).

Surprisingly, no effort has been made so far to develop allosteric activators binding at the ATP site capable of increasing the levels of D-serine for the treatment of schizophrenia, which is characterized by low D-serine concentrations in the brain. This avenue possibly needs to await the full determination of the complex hSR interactome.

## Conformational Landscape of hSR

**Scheme 3:**
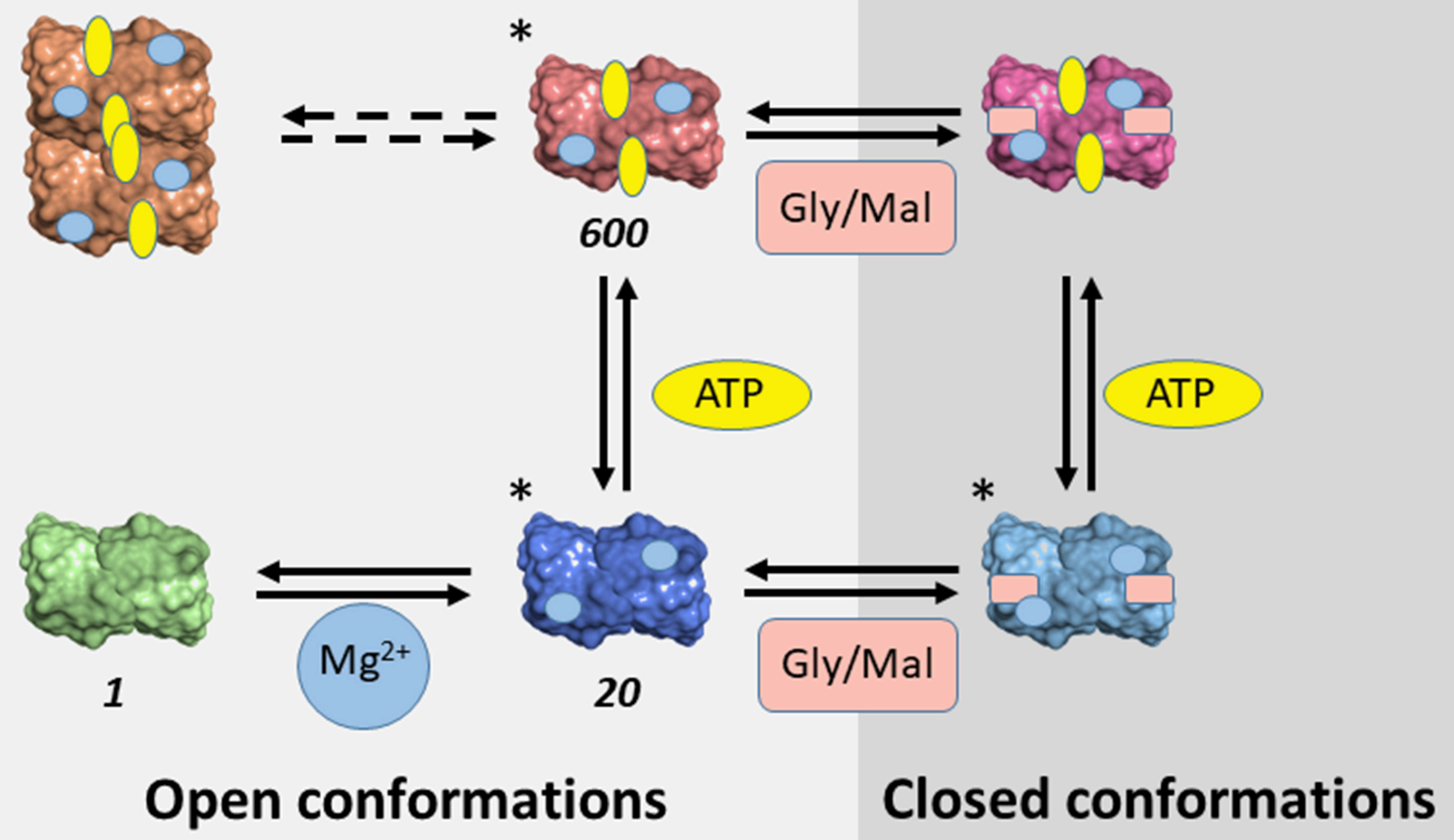
Conformational space of SR. The conformations determined crystallographically are marked with (*). For conformations characterized in terms of catalytic activity, the relative value of catalytic efficiency in comparison with the metal-free form is reported. Gly and Mal indicate glycine and malonate, respectively.

The evidence accumulated on SR suggests that the enzyme is partitioned among different conformations characterized by distinct functional properties depending on the bound ligands (Table 4). Some of these conformations were crystallographically detected and classified as open and closed enzyme states (Scheme 3). The transition involves the reorientation of the small domain with respect to the large one within each subunit, leading to a closure of the active site. Such dynamic events are common to many PLP-dependent enzymes, such as tryptophan synthase, *O*-acetylserine sulfhydrylase and aspartate aminotransferase. Further conformational states, such as the form present in the absence of divalent cations, were inferred to be open-like based on fluorescence experiments (Bruno et al., 2017). A further level of complexity is generated by observations suggesting the existence of equilibria between dimers and tetramers in solution experiments for mSR and hSR (Wang and Barger, 2011; Marchetti et al., 2015; Bruno et al., 2017). The different conformations are associated with significantly different catalytic properties, ranging from the lowest catalytic efficiency in the absence of divalent cations, to the intermediate –20-fold higher–when Mg^2+^ is added, to the highest when ATP is also present, with a further 30-fold increase (Scheme 3). Furthermore, ATP binds cooperatively, with a dissociation constant ranging from millimolar to low micromolar range depending on the ligation state of the active site. Conversely, the affinity of malonate and glycine increased several fold in the presence of ATP.

**Table 4:** SR interactors and effects on SR structure and function.

**Interactors**	**Isoform**	**Site of interaction on SR**	**Effect**	**Experimental evidences**	**References**
**SMALL LIGANDS**					
*ATP*	Human	Dimer interface(by similarity)	Structural rearrangement, activation	Activity assays, binding assays, molecular docking	Jirásková-Vanícková et al., 2011 Marchetti et al., 2013 Marchetti et al., 2015 Canosa et al., 2018
	Mouse	Dimer interface(by similarity)	Activation	Activity assays	Cook et al., 2002 De Miranda et al., 2002 Neidle and Dunlop, 2002
	S. pombe^*^	Dimer interface	Structural rearrangement, activation	Activity assays, X-ray crystallography	Goto et al., 2009
*Mg^2+^/Ca^2+^*	Human^**^	Glu210, Ala214, Asp216	Activation	X-ray crystallography, activity assays	Smith et al., 2010 Bruno et al., 2017
	Mouse	Glu210, Ala214, Asp216(by similarity)	Activation	Activity assays, circular dichroism, radiolabelling	Cook et al., 2002 De Miranda et al., 2002 Neidle and Dunlop, 2002
	Rat^**^	Glu210, Ala214, Asp216	Not determined	X-ray crystallography	Smith et al., 2010
	S. pombe	ATP binding siteGlu208, Gly212, Asp214	Activation	Activity assays, X-ray crystallography	Goto et al., 2009 Yamauchi et al., 2009
*Cl^−^*	Human	Unidentified	Activation, oligomerization	Activity assays, gel filtration	Marchetti et al., 2015 Bruno et al., 2017
*NADH*	Human	Dimer interface	Inhibition	Activity assays	Bruno et al., 2016
*Glycine*	Human	Active site	Inhibition	Activity assays, binding assays	Hoffman et al., 2009 Marchetti et al., 2013
	Mouse	Active site	Inhibition	Activity assays	Dunlop and Neidle, 2005 Strísovský et al., 2005
*Malonate*	Human	Active site	Inhibition	Activity assays, binding assays, X-ray crystallography	Strísovský et al., 2005 Smith et al., 2010 Marchetti et al., 2015
	Rat	Active site	Inhibition	X-ray crystallography	Smith et al., 2010
*PIP_2_*	Mouse	Lys70, Lys77, Lys96, Lys137, Leu168	Inhibition, association to intracellular membranes	Immunocytochemistry, activity assays, binding assays	Mustafa et al., 2009
**POST-TRANSLATIONAL MODIFICATIONS**					
*Nitrosylation*	Human	Cys113, Cys128, Cys269	Inhibition	Activity assays, binding assays, mass spectrometry	Marchesani et al., 2018
	Mouse	Cys113	Inhibition	Activity assays	Shoji et al., 2006 Mustafa et al., 2007
*Phosphorylation*	Mouse	Thr71, Thr227	Activation	Mass spectrometry, radiolabelling, activity assays	Balan et al., 2009 Foltyn et al., 2010
*Acylation/palmitoylation*	Mouse	Unidentified Ser or Thr	Translocation to membranes	Radiolabelling, immunocytochemistry	Balan et al., 2009
*Ubiquitination*	Mouse	Not determined	Degradation	Western blotting	Dumin et al., 2006
**PROTEINS**					
*GRIP*	Mouse	C-terminus	Activation	Western blotting, activity assays	Jirásková-Vanícková et al., 2011
	Rat	C-terminus	Activation	Yeast two-hybrid screening, immunoprecipitation	Kim et al., 2005
*PICK1*	Human	C-terminus	Activation	Yeast two-hybrid screening, binding assays, immunoprecipitation, immunocytochemistry	Fujii et al., 2005
	Mouse	C-terminus	Activation	Binding assays, siRNA, western blotting, immunoprecipitation, HPLC	Hikida et al., 2008 Zhuang et al., 2010
*Stargazin/PSD-95*	Mouse	C-terminus	Inhibition	Immunoprecipitation, western blotting, immunocytochemistry, HPLC	Ma et al., 2014
*Golga-3*	Mouse	N-terminus	Increased half-life	Yeast two-hybrid screening, immunoprecipitation, immunocytochemistry, binding assays	Dumin et al., 2006
*DISC1*	Human	Not determined	Increased half-life	Western blotting, immunoprecipitation, immunocytochemistry	Ma et al., 2013
	Mouse	Not determined	Increased half-life	Immunohistochemistry, western blotting, immunoprecipitation, immunocytochemistry, binding assays	Ma et al., 2013
*FBXO22*	Mouse	Not determined	Prevention of translocation to membranes	Activity assays, binding assays, immunoprecipitation	Dikopoltsev et al., 2014

* *Co-crystallized with the ATP analog AMP-PCP*.

** *Mn^2+^ in the crystallographic structure*.

## Perspectives

Overall, results obtained on hSR indicate that protein function is regulated by alterations of the conformational distribution (Table 4), i.e. effectors stabilize distinct tertiary and quaternary enzyme conformations. This finding might be exploited for the design of positive and negative allosteric effectors with therapeutic actions. However, in order to proceed along this pathway, the three-dimensional structures of hSR in the presence of ligands that bind to either the active site or the allosteric site should be determined. This aim might be challenging due to difficulties in hSR crystallization. Indeed, surprisingly, so far the structure of hSR in the presence of ATP or a stable ATP analog has not yet been determined. As cryo-EM methods for protein structural determination are approaching the resolution of X-ray crystallography, it might be feasible to collect structures of hSR complexed with ligands and protein interactors in order to obtain a more detailed map of the conformational landscape of SR. In turn, this information should improve the identification of hits *in silico* ligand screening campaigns.

## Author Contributions

SR, MMarc, SF, BC, SB, MMarg, FM and AM contributed by reviewing the literature and writing distinct sections of the manuscript. Specifically, SR and SB focused on PTM, SF on structure, BC on catalysis, MMarc and MMarg on effectors, AM and FM on inhibitors. AM designed the manuscript and coordinated the activities. All authors read and approved the manuscript.

### Conflict of Interest Statement

The authors declare that the research was conducted in the absence of any commercial or financial relationships that could be construed as a potential conflict of interest.

## References

[B1] Balan L. Foltyn V. N. Zehl M. Dumin E. Dikopoltsev E. Knoh D.. (2009). Feedback inactivation of D-serine synthesis by NMDA receptor-elicited translocation of serine racemase to the membrane. Proc. Natl. Acad. Sci. U.S.A. 106, 7589–7594. 10.1073/pnas.0809442106 19380732 PMC2678608

[B2] Balu D. T. Li Y. Puhl M. D. Benneyworth M. A. Basu A. C. Takagi S.. (2013). Multiple risk pathways for schizophrenia converge in serine racemase knockout mice, a mouse model of NMDA receptor hypofunction. Proc. Natl. Acad. Sci. U.S.A. 110, E2400–E2409. 10.1073/pnas.1304308110 23729812 PMC3696825

[B3] Baumgart F. Mancheño J. M. Rodríguez-Crespo I. (2007). Insights into the activation of brain serine racemase by the multi-PDZ domain glutamate receptor interacting protein, divalent cations and ATP. FEBS J. 274, 4561–4571. 10.1111/j.1742-4658.2007.05986.x 17697119

[B4] Beato C. Pecchini C. Cocconcelli C. Campanini B. Marchetti M. Pieroni M.. (2016). Cyclopropane derivatives as potential human serine racemase inhibitors: unveiling novel insights into a difficult target. J. Enzyme Inhib. Med. Chem. 31, 645–652. 10.3109/14756366.2015.1057720 26133542

[B5] Bettati S. Benci S. Campanini B. Raboni S. Chirico G. Beretta S.. (2000). Role of pyridoxal 5′-phosphate in the structural stabilization of O-acetylserine sulfhydrylase. J. Biol. Chem. 275, 40244–40251. 10.1074/jbc.M007015200 10995767

[B6] Bruno S. Marchesani F. Dellafiora L. Margiotta M. Faggiano S. Campanini B.. (2016). Human serine racemase is allosterically modulated by NADH and reduced nicotinamide derivatives. Biochem. J. 473, 3505–3516. 10.1042/BCJ20160566 27493223

[B7] Bruno S. Margiotta M. Marchesani F. Paredi G. Orlandi V. Faggiano S.. (2017). Magnesium and calcium ions differentially affect human serine racemase activity and modulate its quaternary equilibrium toward a tetrameric form. Biochim. Biophys. Acta 1865, 381–387. 10.1016/j.bbapap.2017.01.001 28089597

[B8] Bruno S. Schiaretti F. Burkhard P. Kraus J. P. Janosik M. Mozzarelli A. (2001). Functional properties of the active core of human cystathionine beta-synthase crystals. J. Biol. Chem. 276, 16–19. 10.1074/jbc.C000588200 11042162

[B9] Campanini B. Raboni S. Vaccari S. Zhang L. Cook P. F. Hazlett T. L.. (2003). Surface-exposed tryptophan residues are essential for O-acetylserine sulfhydrylase structure, function, and stability. J. Biol. Chem. 278, 37511–37519. 10.1074/jbc.M305138200 12813039

[B10] Canosa A. V. Faggiano S. Marchetti M. Armao S. Bettati S. Bruno S.. (2018). Glutamine 89 is a key residue in the allosteric modulation of human serine racemase activity by ATP. Sci. Rep. 8, 9016. 10.1038/s41598-018-27227-1 29899358 PMC5998037

[B11] Canu N. Ciotti M. T. Pollegioni L. (2014). Serine racemase: a key player in apoptosis and necrosis. Front. Synaptic Neurosci. 6:9. 10.3389/fnsyn.2014.00009 24795622 PMC4000995

[B12] Conti P. Tamborini L. Pinto A. Blondel A. Minoprio P. Mozzarelli A.. (2011). Drug discovery targeting amino acid racemases. Chem. Rev. 111, 6919–6946. 10.1021/cr2000702 21913633

[B13] Cook S. P. Galve-Roperh I. Martínez del Pozo A. Rodríguez-Crespo I. (2002). Direct calcium binding results in activation of brain serine racemase. J. Biol. Chem. 277, 27782–27792. 10.1074/jbc.M111814200 12021263

[B14] Cooper A. J. Bruschi S. A. Iriarte A. Martinez-Carrion M. (2002). Mitochondrial aspartate aminotransferase catalyses cysteine S-conjugate beta-lyase reactions. Biochem. J. 368, 253–261. 10.1042/bj20020531 12137566 PMC1222959

[B15] De Miranda J. Panizzutti R. Foltyn V. N. Wolosker H. (2002). Cofactors of serine racemase that physiologically stimulate the synthesis of the N-methyl-D-aspartate (NMDA) receptor coagonist D-serine. Proc. Natl. Acad. Sci. U.S.A. 99, 14542–14547. 10.1073/pnas.222421299 12393813 PMC137919

[B16] De Miranda J. Santoro A. Engelender S. Wolosker H. (2000). Human serine racemase: moleular cloning, genomic organization and functional analysis. Gene 256, 183–188. 10.1016/S0378-1119(00)00356-5 11054547

[B17] Dellafiora L. Marchetti M. Spyrakis F. Orlandi V. Campanini B. Cruciani G.. (2015). Expanding the chemical space of human serine racemase inhibitors. Bioorg. Med. Chem. Lett. 25, 4297–4303. 10.1016/j.bmcl.2015.07.081 26283510

[B18] Dikopoltsev E. Foltyn V. N. Zehl M. Jensen O. N. Mori H. Radzishevsky I.. (2014). FBXO22 protein is required for optimal synthesis of the N-methyl-D-aspartate (NMDA) receptor coagonist D-serine. J. Biol. Chem. 289, 33904–33915. 10.1074/jbc.M114.618405 25336657 PMC4256329

[B19] Dixon S. M. Li P. Liu R. Wolosker H. Lam K. S. Kurth M. J.. (2006). Slow-binding human serine racemase inhibitors from high-throughput screening of combinatorial libraries. J. Medl. Chem. 49, 2388–2397. 10.1021/jm050701c 16610782

[B20] Doyon N. Vinay L. Prescott S. A. De Koninck Y. (2016). Chloride regulation: a dynamic equilibrium crucial for synaptic inhibition. Neuron 89, 1157–1172. 10.1016/j.neuron.2016.02.030 26985723

[B21] Dumin E. Bendikov I. Foltyn V. N. Misumi Y. Ikehara Y. Kartvelishvily E.. (2006). Modulation of D-serine levels via ubiquitin-dependent proteasomal degradation of serine racemase. J. Biol. Chem. 281, 20291–20302. 10.1074/jbc.M601971200 16714286

[B22] Dunlop D. S. Neidle A. (2005). Regulation of serine racemase activity by amino acids. Brain Res. Mol. Brain Res. 133, 208–214. 10.1016/j.molbrainres.2004.10.027 15710237

[B23] Foltyn V. N. Bendikov I. De Miranda J. Panizzutti R. Dumin E. Shleper M.. (2005). Serine racemase modulates intracellular D-serine levels through an alpha,beta-elimination activity. J. Biol. Chem. 280, 1754–1763. 10.1074/jbc.M405726200 15536068

[B24] Foltyn V. N. Zehl M. Dikopoltsev E. Jensen O. N. Wolosker H. (2010). Phosphorylation of mouse serine racemase regulates D-serine synthesis. FEBS Lett. 584, 2937–2941. 10.1016/j.febslet.2010.05.022 20493854

[B25] Fujii K. Maeda K. Hikida T. Mustafa A. K. Balkissoon R. Xia J.. (2005). Serine racemase binds to PICK1: potential relevance to schizophrenia. Mol. Psychiatry 11, 150–157. 10.1038/sj.mp.4001776 16314870

[B26] Genc S. Kurnaz I. A. Ozilgen M. (2011). Astrocyte - neuron lactate shuttle may boost more ATP supply to the neuron under hypoxic conditions - *in silico* study supported by *in vitro* expression data. BMC Syst. Biol. 5:162. 10.1186/1752-0509-5-162 21995951 PMC3202240

[B27] Goto M. Yamauchi T. Kamiya N. Miyahara I. Yoshimura T. Mihara H.. (2009). Crystal structure of a homolog of mammalian serine racemase from Schizosaccharomyces pombe. J. Biol. Chem. 284, 25944–25952. 10.1074/jbc.M109.010470 19640845 PMC2757995

[B28] Gribble F. M. Loussouarn G. Tucker S. J. Zhao C. Nichols C. G. Ashcroft F. M. (2000). A novel method for measurement of submembrane ATP concentration. J. Biol. Chem. 275, 30046–30049. 10.1074/jbc.M001010200 10866996

[B29] Grishin N. V. Phillips M. A. Goldsmith E. J. (1995). Modeling of the spatial structure of eukaryotic ornithine decarboxylases. Protein Sci. 4, 1291–1304. 10.1002/pro.5560040705 7670372 PMC2143167

[B30] Griswold W. R. Toney M. D. (2011). Role of the pyridine nitrogen in pyridoxal 5′-phosphate catalysis: activity of three classes of PLP enzymes reconstituted with deazapyridoxal 5′-phosphate. J. Am. Chem. Soc. 133, 14823–14830. 10.1021/ja2061006 21827189

[B31] Hanley J. G. (2008). PICK1: A multi-talented modulator of AMPA receptor trafficking. Pharmacol. Ther. 118, 152–160. 10.1016/j.pharmthera.2008.02.002 18353440

[B32] Hashimoto A. Nishikawa T. Hayashi T. Fujii N. Harada K. Oka T.. (1992). The presence of free D-serine in rat brain. FEBS Lett. 296, 33–36. 10.1016/0014-5793(92)80397-Y 1730289

[B33] Hikida T. Mustafa A. K. Maeda K. Fujii K. Barrow R. K. Saleh M.. (2008). Modulation of D-serine levels in brains of mice lacking PICK1. Biol. Psychiatry 63, 997–1000. 10.1016/j.biopsych.2007.09.025 18191108 PMC2715963

[B34] Hoffman H. E. Jirásková J. Cígler P. Sanda M. Schraml J. Konvalinka J. (2009). Hydroxamic acids as a novel family of serine racemase inhibitors: mechanistic analysis reveals different modes of interaction with the pyridoxal-5′-phosphate cofactor. J. Med. Chem. 52, 6032–6041. 10.1021/jm900775q 19791805

[B35] Ito T. Hayashida M. Kobayashi S. Muto N. Hayashi A. Yoshimura T.. (2016). Serine racemase is involved in D-aspartate biosynthesis. J. Biochem. 160, 345–353. 10.1093/jb/mvw043 27387750

[B36] Ito T. Murase H. Maekawa M. Goto M. Hayashi S. Saito H.. (2012). Metal ion dependency of serine racemase from Dictyostelium discoideum. Amino Acids 43, 1567–1576. 10.1007/s00726-012-1232-z 22311068

[B37] Jäger J. Moser M. Sauder U. Jansonius J. N. (1994). Crystal structures of Escherichia coli aspartate aminotransferase in two conformations. Comparison of an unliganded open and two liganded closed forms. J. Mol. Biol. 239, 285–305. 10.1006/jmbi.1994.1368 8196059

[B38] Jansonius J. N. (1998). Structure, evolution and action of vitamin B6-dependent enzymes. Curr. Opin. Struct. Biol. 8, 759–769. 10.1016/S0959-440X(98)80096-1 9914259

[B39] Jansonius J. N. Vincent M. G. (1987). Structural basis for catalysis of aspartate aminotransferase, in Biological Macromolecules and Assemblies, eds Jurnak F. A. Mcpherson A. (New York, NY: J. Wiley & Sons, Inc.), 187–285.

[B40] Jirásková-Vanícková J. Ettrich R. Vorlová B. Hoffman H. E. Lepšík M. Jansa P.. (2011). Inhibition of human serine racemase, an emerging target for medicinal chemistry. Curr. Drug Targets 12, 1037–1055. 10.2174/138945011795677755 21291385

[B41] Kaiser J. T. Bruno S. Clausen T. Huber R. Schiaretti F. Mozzarelli A.. (2003). Snapshots of the cystine lyase C-DES during catalysis. Studies in solution and in the crystalline state. J. Biol. Chem. 278, 357–365. 10.1074/jbc.M209862200 12386155

[B42] Kim P. M. Aizawa H. Kim P. S. Huang A. S. Wickramasinghe S. R. Kashani A. H.. (2005). Serine racemase: activation by glutamate neurotransmission via glutamate receptor interacting protein and mediation of neuronal migration. Proc. Natl. Acad. Sci. U.S.A. 102, 2105–2110. 10.1073/pnas.0409723102 15684087 PMC548584

[B43] Kiriyama Y. Nochi H. (2016). D-amino acids in the nervous and endocrine systems. Scientifica (Cairo) 2016:6494621. 10.1155/2016/6494621 28053803 PMC5178360

[B44] Labrie V. Fukumura R. Rastogi A. Fick L. J. Wang W. Boutros P. C.. (2009). Serine racemase is associated with schizophrenia susceptibility in humans and in a mouse model. Hum. Mol. Genet. 18, 3227–3243. 10.1093/hmg/ddp261 19483194 PMC2722985

[B45] Lugo-Huitrón R. Ugalde Muñiz P. Pineda B. Pedraza-Chaverrí J. Ríos C. Perez-De La Cruz V. (2013). Quinolinic acid: an endogenous neurotoxin with multiple targets. Oxid. Med. Cell. Longev. 2013:104024. 10.1155/2013/104024 24089628 PMC3780648

[B46] Ma T. M. Abazyan S. Abazyan B. Nomura J. Yang C. Seshadri S.. (2013). Pathogenic disruption of DISC1-serine racemase binding elicits schizophrenia-like behavior via D-serine depletion. Mol. Psychiatry 18, 557–567. 10.1038/mp.2012.97 22801410 PMC3475769

[B47] Ma T. M. Paul B. D. Fu C. Hu S. Zhu H. Blackshaw S.. (2014). Serine racemase regulated by binding to stargazin and PSD-95: potential N-methyl-D-aspartate-alpha-amino-3-hydroxy-5-methyl-4-isoxazolepropionic acid (NMDA-AMPA) glutamate neurotransmission cross-talk. J. Biol. Chem. 289, 29631–29641. 10.1074/jbc.M114.571604 25164819 PMC4207978

[B48] Marchesani F. Bruno S. Paredi G. Raboni S. Campanini B. Mozzarelli A. (2018). Human serine racemase is nitrosylated at multiple sites. Biochim. Biophys. Acta 1866, 813–821. 10.1016/j.bbapap.2018.01.009 29410194

[B49] Marchetti M. Bruno S. Campanini B. Bettati S. Peracchi A. Mozzarelli A. (2015). Regulation of human serine racemase activity and dynamics by halides, ATP and malonate. Amino Acids 47, 163–173. 10.1007/s00726-014-1856-2 25331425

[B50] Marchetti M. Bruno S. Campanini B. Peracchi A. Mai N. Mozzarelli A. (2013). ATP binding to human serine racemase is cooperative and modulated by glycine. FEBS J. 280, 5853–5863. 10.1111/febs.12510 23992455

[B51] Maruyama K. (1990). Activation of pseudomonas ochraceae 4-hydroxy-4-methyl-2-oxoglutarate aldolase by inorganic phosphate. J. Bioch. 108, 334–340. 10.1093/oxfordjournals.jbchem.a123202 2229033

[B52] Mehta P. K. Christen P. (2000). The molecular evolution of pyridoxal-5′-phosphate-dependent enzymes. Adv. Enzymol. Relat. Areas Mol. Biol. 74, 129–184. 10.1002/9780470123201.ch4 10800595

[B53] Mori H. Wada R. Li J. Ishimoto T. Mizuguchi M. Obita T.. (2014). *In silico* and pharmacological screenings identify novel serine racemase inhibitors. Bioorg. Med. Chem. Lett. 24, 3732–3735. 10.1016/j.bmcl.2014.07.003 25066953

[B54] Mori H. Wada R. Takahara S. Horino Y. Izumi H. Ishimoto T.. (2017). A novel serine racemase inhibitor suppresses neuronal over-activation *in vivo*. Bioorg. Med. Chem. 25, 3736–3745. 10.1016/j.bmc.2017.05.011 28533113

[B55] Morino Y. Kojima H. Tanase S. (1979). Affinity labeling of alanine aminotransferase by 3-chloro-L-alanine. J. Biol. Chem. 254, 279–285. 33167

[B56] Mozzarelli A. Bettati S. Campanini B. Salsi E. Raboni S. Singh R.. (2011). The multifaceted pyridoxal 5′-phosphate-dependent O-acetylserine sulfhydrylase. Biochim. Biophys. Acta 1814, 1497–1510. 10.1016/j.bbapap.2011.04.011 21549222

[B57] Mustafa A. K. Kim P. M. Snyder S. H. (2004). D-Serine as a putative glial neurotransmitter. Neuron Glia Biol. 1, 275–281. 10.1017/S1740925X05000141 16543946 PMC1403160

[B58] Mustafa A. K. Kumar M. Selvakumar B. Ho G. P. Ehmsen J. T. Barrow R. K.. (2007). Nitric oxide S-nitrosylates serine racemase, mediating feedback inhibition of D-serine formation. Proc. Natl. Acad. Sci. U.S.A. 104, 2950–2955. 10.1073/pnas.0611620104 17293453 PMC1815287

[B59] Mustafa A. K. van Rossum D. B. Patterson R. L. Maag D. Ehmsen J. T. Gazi S. K.. (2009). Glutamatergic regulation of serine racemase via reversal of PIP2 inhibition. Proc. Natl. Acad. Sci. U.S.A. 106, 2921–2926. 10.1073/pnas.0813105106 19193859 PMC2635840

[B60] Neidle A. Dunlop D. S. (2002). Allosteric regulation of mouse brain serine racemase. Neurochem. Res. 27, 1719–1724. 10.1023/A:1021607715824 12515328

[B61] Németh H. Toldi J. Vécsei L. (2006). Kynurenines, Parkinson's disease and other neurodegenerative disorders: preclinical and clinical studies. J. Neural Transm. Suppl. 70, 285–304. 10.1007/978-3-211-45295-0_45 17017544

[B62] Ohnishi M. Saito M. Wakabayashi S. Ishizuka M. Nishimura K. Nagata Y.. (2008). Purification and characterization of serine racemase from a hyperthermophilic archaeon, Pyrobaculum islandicum. J. Bacteriol. 190, 1359–1365. 10.1128/JB.01184-07 17965169 PMC2238205

[B63] Okamoto A. Higuchi T. Hirotsu K. Kuramitsu S. Kagamiyama H. (1994). X-ray crystallographic study of pyridoxal 5′-phosphate-type aspartate aminotransferases from *Escherichia coli* in open and closed form. J. Biochem. 116, 95–107. 10.1093/oxfordjournals.jbchem.a124509 7798192

[B64] Panizzutti R. De Miranda J. Ribeiro C. S. Engelender S. Wolosker H. (2001). A new strategy to decrease N-methyl-D-aspartate (NMDA) receptor coactivation: inhibition of D-serine synthesis by converting serine racemase into an eliminase. Proc. Natl. Acad. Sci. USA. 98, 5294–5299. 10.1073/pnas.091002298 11309496 PMC33203

[B65] Paoletti P. Bellone C. Zhou Q. (2013). NMDA receptor subunit diversity: impact on receptor properties, synaptic plasticity and disease. Nat. Rev. Neurosci. 14, 383–400. 10.1038/nrn3504 23686171

[B66] Percudani R. Peracchi A. (2009). The B6 database: a tool for the description and classification of vitamin B6-dependent enzymatic activities and of the corresponding protein families. BMC Bioinformatics 10:273. 10.1186/1471-2105-10-273 19723314 PMC2748086

[B67] Phillips R. S. Demidkina T. V. Zakomirdina L. N. Bruno S. Ronda L. Mozzarelli A. (2002). Crystals of tryptophan indole-lyase and tyrosine phenol-lyase form stable quinonoid complexes. J. Biol. Chem. 277, 21592–21597. 10.1074/jbc.M200216200 11934889

[B68] Price G. D. Trussell L. O. (2006). Estimate of the chloride concentration in a central glutamatergic terminal: a gramicidin perforated-patch study on the calyx of Held. J. Neurosci. 26, 11432–11436. 10.1523/JNEUROSCI.1660-06.2006 17079672 PMC6674540

[B69] Purich D. Allison R. (2002). The Enzyme Reference: A Comprehensive Guidebook to Enzyme Nomenclature, Reactions, and Methods. San Diego, CA: Academic Press.

[B70] Raboni S. Bettati S. Mozzarelli A. (2005). Identification of the geometric requirements for allosteric communication between the alpha- and beta-subunits of tryptophan synthase. J. Biol. Chem. 280, 13450–13456. 10.1074/jbc.M414521200 15691828

[B71] Raboni S. Bettati S. Mozzarelli A. (2009). Tryptophan synthase: a mine for enzymologists. Cell. Mol. Life Sci. 66, 2391–2403. 10.1007/s00018-009-0028-0 19387555 PMC11115766

[B72] Raboni S. Mozzarelli A. Cook P. F. (2007). Control of ionizable residues in the catalytic mechanism of tryptophan synthase from Salmonella typhimurium. Biochemistry 46, 13223–13234. 10.1021/bi701152f 17927213

[B73] Raboni S. Pioselli B. Bettati S. Mozzarelli A. (2003). The molecular pathway for the allosteric regulation of tryptophan synthase. Biochim. Biophys. Acta 1647, 157–160. 10.1016/S1570-9639(03)00084-0 12686126

[B74] Raboni S. Spyrakis F. Campanini B. Amadasi A. Bettati S. Peracchi A. (2010). Pyridoxal 5-phosphate-dependent enzymes: catalysis, conformation, and genomics, in Comprehensive Natural Products II: Chemistry and Biology., eds Mander L. Liu H.-W. (Oxford: Elsevier), 273–315.

[B75] Schneider G. Käck H. Lindqvist Y. (2000). The manifold of vitamin B6 dependent enzymes. Structure 8, R1–R6. 10.1016/S0969-2126(00)00085-X 10673430

[B76] Shoji K. Mariotto S. Ciampa A. R. Suzuki H. (2006). Regulation of serine racemase activity by D-serine and nitric oxide in human glioblastoma cells. Neurosci. Lett. 392, 75–78. 10.1016/j.neulet.2005.08.063 16182447

[B77] Smith M. A. Mack V. Ebneth A. Moraes I. Felicetti B. Wood M.. (2010). The structure of mammalian Serine Racemase: evidence for conformational changes upon inhibitor binding. J. Biol. Chem. 285, 12873–12881. 10.1074/jbc.M109.050062 20106978 PMC2857111

[B78] Spyrakis F. Cellini B. Bruno S. Benedetti P. Carosati E. Cruciani G.. (2014). Targeting cystalysin, a virulence factor of treponema denticola-supported periodontitis. ChemMedChem 9, 1501–1511. 10.1002/cmdc.201300527 24616267

[B79] Spyrakis F. Felici P. Bayden A. S. Salsi E. Miggiano R. Kellogg G. E.. (2013). Fine tuning of the active site modulates specificity in the interaction of O-acetylserine sulfhydrylase isozymes with serine acetyltransferase. Biochim. Biophys. Acta 1834, 169–181. 10.1016/j.bbapap.2012.09.009 23000429

[B80] Spyrakis F. Raboni S. Cozzini P. Bettati S. Mozzarelli A. (2006). Allosteric communication between alpha and beta subunits of tryptophan synthase: modelling the open-closed transition of the alpha subunit. Biochim. Biophys. Acta 1764, 1102–1109. 10.1016/j.bbapap.2006.03.005 16737856

[B81] Storici P. De Biase D. Bossa F. Bruno S. Mozzarelli A. Peneff C. (2004). Structures of gamma-aminobutyric acid (GABA) aminotransferase, a pyridoxal 5′-phosphate, and [2Fe-2S] cluster-containing enzyme, complexed with gamma-ethynyl-GABA and with the antiepilepsy drug vigabatrin. J. Biol. Chem. 279, 363–373. 10.1074/jbc.M305884200 14534310

[B82] Strísovský K. Jirásková J. Mikulová A. Rulísek L. Konvalinka J. (2005). Dual substrate and reaction specificity in mouse serine racemase: identification of high-affinity dicarboxylate substrate and inhibitors and analysis of the beta-eliminase activity. Biochemistry 44, 13091–13100. 10.1021/bi051201o 16185077

[B83] Suzuki M. Sasabe J. Miyoshi Y. Kuwasako K. Muto Y. Hamase K.. (2015). Glycolytic flux controls D-serine synthesis through glyceraldehyde-3-phosphate dehydrogenase in astrocytes. Proc. Natl. Acad. Sci. U.S.A. 112, E2217–2224. 10.1073/pnas.1416117112 25870284 PMC4418896

[B84] Takahara S. Nakagawa K. Uchiyama T. Yoshida T. Matsumoto K. Kawasumi Y. (2018). Design, synthesis, and evaluation of novel inhibitors for wild-type human serine racemase. Bioorg. Med. Chem. Lett. 28, 441–445. 10.1016/j.bmcl.2017.12.021 29277459

[B85] Toney M. D. (2005). Reaction specificity in pyridoxal phosphate enzymes. Arch. Biochem. Biophys. 433, 279–287. 10.1016/j.abb.2004.09.037 15581583

[B86] Uda K. Abe K. Dehara Y. Mizobata K. Edashige Y. Nishimura R.. (2017). Triple serine loop region regulates the aspartate racemase activity of the serine/aspartate racemase family. Amino Acids 49, 1743–1754. 10.1007/s00726-017-2472-8 28744579

[B87] Uda K. Abe K. Dehara Y. Mizobata K. Sogawa N. Akagi Y.. (2016). Distribution and evolution of the serine/aspartate racemase family in invertebrates. Amino Acids 48, 387–402. 10.1007/s00726-015-2092-0 26352274

[B88] Vargas-Lopes C. Madeira C. Kahn S. A. Albino Do Couto I. Bado P. Houzel J. C.. (2011). Protein kinase C activity regulates D-serine availability in the brain. J. Neurochem. 116, 281–290. 10.1111/j.1471-4159.2010.07102.x 21070240

[B89] Vorlová B. Nachtigallová D. Jirásková-Vanícková J. Ajani H. Jansa P. Rezác J.. (2015). Malonate-based inhibitors of mammalian serine racemase: kinetic characterization and structure-based computational study. Eur. J. Med. Chem. 89, 189–197. 10.1016/j.ejmech.2014.10.043 25462239

[B90] Wang W. Barger S. W. (2011). Roles of quaternary structure and cysteine residues in the activity of human serine racemase. BMC Biochem. 12:63. 10.1186/1471-2091-12-63 22151352 PMC3252284

[B91] Wild J. R. Belser W. L. O'donovan G. A. (1976). Unique aspects of the regulation of the aspartate transcarbamylase of Serratia marcescens. J. Bacteriol. 128, 766–775. 11207 10.1128/jb.128.3.766-775.1976 PMC232767

[B92] Wolosker H. Balu D. T. Coyle J. T. (2016). The rise and fall of the D-serine-mediated gliotransmission hypothesis. Trends Neurosci. 39, 712–721. 10.1016/j.tins.2016.09.007 27742076 PMC5113294

[B93] Wolosker H. Sheth K. N. Takahashi M. Mothet J. P. Brady R. O. Ferris C. D.. (1999). Purification of serine racemase: biosynthesis of the neuromodulator D-serine. Proc. Natl. Acad. Sci. U.S.A. 96, 721–725. 10.1073/pnas.96.2.721 9892700 PMC15203

[B94] Yamauchi T. Goto M. Wu H. Y. Uo T. Yoshimura T. Mihara H.. (2009). Serine racemase with catalytically active lysinoalanyl residue. J. Biochem. 145, 421–424. 10.1093/jb/mvp010 19155267

[B95] Yoshimura T. Esak N. (2003). Amino acid racemases: functions and mechanisms. J. Biosci. Bioeng. 96, 103–109. 10.1016/S1389-1723(03)90111-3 16233494

[B96] Yoshimura T. Goto M. (2008). D-amino acids in the brain: structure and function of pyridoxal phosphate-dependent amino acid racemases. FEBS J. 275, 3527–3537. 10.1111/j.1742-4658.2008.06516.x 18564179

[B97] Zhuang Z. Yang B. Theus M. H. Sick J. T. Bethea J. R. Sick T. J.. (2010). EphrinBs regulate D-serine synthesis and release in astrocytes. J. Neurosci. 30, 16015–16024. 10.1523/JNEUROSCI.0481-10.2010 21106840 PMC3073557

[B98] Zou L. Song Y. Wang C. Sun J. Wang L. Cheng B.. (2016). Crystal structure of maize serine racemase with pyridoxal 5′-phosphate. Acta Crystallogr. F. 72, 165–171. 10.1107/S2053230X16000960 26919519 PMC4774874

